# Methods for joint modeling of longitudinal omics data and time-to-event outcomes: applications to lysophosphatidylcholines in connection to aging and mortality in the Long Life Family Study

**DOI:** 10.18632/aging.206259

**Published:** 2025-05-27

**Authors:** Konstantin G. Arbeev, Olivia Bagley, Svetlana V. Ukraintseva, Alexander Kulminski, Eric Stallard, Michaela Schwaiger-Haber, Gary J. Patti, Yian Gu, Anatoliy I. Yashin, Michael A. Province

**Affiliations:** 1 Biodemography of Aging Research Unit, Social Science Research Institute, Duke University, Durham, NC 27708, USA; 2 Department of Chemistry, Washington University in St. Louis, St. Louis, MO 63130, USA; 3 Department of Medicine, Washington University in St. Louis, St. Louis, MO 63130, USA; 4 Center for Metabolomics and Isotope Tracing at Washington University in St. Louis, St. Louis, MO 63130, USA; 5 Taub Institute for Research on Alzheimer’s Disease and the Aging Brain, Vagelos College of Physicians and Surgeons, Columbia University, New York, NY 10032, USA; 6 G.H. Sergievsky Center, Vagelos College of Physicians and Surgeons, Columbia University, New York, NY 10032, USA; 7 Department of Neurology, Vagelos College of Physicians and Surgeons, Columbia University, and the New York Presbyterian Hospital, New York, NY 10032, USA; 8 Department of Epidemiology, Mailman School of Public Health, Columbia University, New York, NY 10032, USA; 9 Division of Statistical Genomics, Department of Genetics, Washington University School of Medicine, St. Louis, MO 63110, USA

**Keywords:** lysophosphatidylcholines, aging, mortality, longitudinal omics, repeated measurements

## Abstract

Studying the relationships between longitudinal changes in omics variables and event risks requires specific methodologies for joint analyses of longitudinal and time-to-event outcomes. We applied two such approaches (joint models [JM], stochastic process models [SPM]) to longitudinal metabolomics data from the Long Life Family Study, focusing on the understudied associations of longitudinal changes in lysophosphatidylcholines (LPCs) with mortality and aging-related outcomes. We analyzed 23 LPC species, with 5,066 measurements of each in 3,462 participants, 1,245 of whom died during follow-up. JM analyses found that higher levels of the majority of LPC species were associated with lower mortality risks, with the largest magnitude observed for LPC 15:0/0:0 (hazard ratio: 0.71, 95% CI (0.64, 0.79)). SPM applications to LPC 15:0/0:0 revealed that the JM association reflects underlying aging-related processes: a decline in robustness to deviations from optimal LPC levels, higher equilibrium LPC levels in females, and the opposite age-related changes in the equilibrium and optimal LPC levels (declining and increasing, respectively), which lead to increased mortality risks with age. Our results support LPCs as biomarkers of aging and related decline in biological robustness, and call for further exploration of factors underlying age-related changes in LPC in relation to mortality and diseases.

## INTRODUCTION

Contemporary longitudinal studies on humans started collecting repeated measurements of various omics (e.g., metabolomics, proteomics) data for study participants. The availability of other types of information on the participants, such as follow-up data on mortality and the onset of diseases, genetic markers, questionnaires, repeated measures of health-related biomarkers, etc., provides extensive opportunities to study complex relations of individual age-trajectories of omics variables with risks of diseases and mortality, in connection to various genetic and non-genetic factors. However, this abundance of information and opportunities comes along with many methodological challenges related to the analyses of such massive data. One particular complication deals with the inherent complexity of analyzing trajectories of health-related variables (repeated measurements of omics variables provide a good example of such) in relation to time-to-event outcomes. The Cox model with time-dependent covariates [1] is the conventional approach traditionally used for joint analyses of time-to-event data and repeated measurements of covariates. However, it is well known that it has certain limitations: ignoring measurement errors or biological variation of covariates and using their observed “raw” values as time-dependent covariates in the Cox model may lead to biased estimates and incorrect inferences [2–4], especially when covariates are measured at sparse examinations or with a long-time interval before an outcome event. This applies to analyzing repeated omics measurements in relation to time-to-event outcomes as well. Even though relevant biostatistical methods, known as joint models (JM) [4, 5], have found broad applications in different research areas, their use in the analyses of longitudinally measured omics data is still limited to a few small-sample proteomics studies [6–8].

One particular class of models for joint analyses of longitudinal and time-to-event outcomes, the stochastic process model (SPM), has been developed in the biodemographic literature based on the mathematical foundations laid out in [9–11]. Recent developments in SPM methodology merged the statistical rigor of the general approach with the biological soundness of specific assumptions built into its structure [12–15] (see [16] for a non-technical introduction to SPM). This brings the biological content to the model structure, making such models particularly appealing for research on aging. The main advantage of using SPM for research on aging is that it allows disentangling a general association between the longitudinal and time-to-event outcomes that can be found using JM into several components representing specific aging-related characteristics embedded in the model. This allows researchers not only to evaluate mortality or incidence rates but also to estimate age-related changes in the mechanism of homeostatic regulation of biological variables, the age-related decline in adaptive capacity and stress resistance, effects of allostatic adaptation, and allostatic load. This provides a more detailed perspective on the impact of the longitudinal changes of the respective variables on the risk of the modeled events in the context of aging. Despite broad applications of SPM to different outcomes and biomarkers (see, e.g., [13, 17–23]), to date, there have been no applications of SPM to analyses of longitudinal omics measurements in relation to time-to-event outcomes.

In this paper, we fill these gaps and apply both JM and SPM to longitudinal measurements of metabolomics collected from participants of the Long Life Family Study (LLFS) [24]. To illustrate applications of the approaches, we focus on a particular class of lipid metabolites, lysophosphatidylcholines (LPCs), which have been actively discussed in the literature in relation to cardiovascular, infectious, and neurodegenerative diseases, and tested as potential early markers of Alzheimer’s disease and accelerated aging [25–31]. Overall, the literature suggests (see, e.g., the recent review [32]) that the reported LPC findings are somewhat contradictory because most of the recent studies, in contrast to older ones, found lower LPC levels to be associated with unfavorable outcomes such as mortality. In addition, the longitudinal changes of LPC in relation to mortality and aging-related outcomes remain understudied. Here, we aimed to test general associations of different LPC species with total (all-cause) mortality in the LLFS using JM and to investigate how such general associations can be decomposed into relations of the mortality risk with different aging-related characteristics (such as robustness, resilience, age-specific norms, and allostatic trajectories [16]), and whether such relationships/characteristics differ by sex.

## RESULTS

### Applications of the basic JM

#### Summary of results

Table 1 shows results of applications of the basic JM [4, 5, 33] (see Eqs. 1–2 in the section Joint models: General specifications in Materials and Methods) to measurements of LPC species and mortality data in the LLFS. The table presents the values of the association parameter (α in Eq. 1) (columns Alpha) for the respective metabolites in the survival sub-model, along with corresponding hazard ratios (for a unit increase in transformed metabolite values) and their 95% confidence intervals (CI) (columns HR (95% CI)) estimated from the JM adjusted for the covariates indicated in the section Joint models: Specific versions used in applications in Materials and Methods. For the majority of LPC species (18 out of 23) in the total sample, the estimates of the association parameter α are negative and CI for respective HR do not contain one. As explained in the section Joint models: General specifications in Materials and Methods, this means that the increase in the levels of these metabolites reduces mortality risk. Sex-specific analyses revealed similar observations for 10 out of 23 LPCs in females and 17 out of 23 LPCs in males.

**Table 1:** Results of applications of joint models to measurements of LPC species and mortality data in the LLFS: Estimates of the association parameter for the metabolite in the survival sub-model.

**Metabolite**	**Total**		**Females**		**Males**	
	**Alpha**	**HR (95% CI)**	**Alpha**	**HR (95% CI)**	**Alpha**	**HR (95% CI)**
LPC 0:0/16:0	−0.185	**0.831 (0.737, 0.938)**	−0.177	**0.838 (0.707, 0.994)**	−0.228	**0.796 (0.665, 0.952)**
LPC 0:0/16:1	−0.036	0.965 (0.849, 1.097)	0.005	1.005 (0.828, 1.220)	−0.046	0.955 (0.803, 1.135)
LPC 0:0/18:0	−0.03	0.970 (0.883, 1.066)	0.005	1.005 (0.880, 1.147)	−0.062	0.940 (0.818, 1.080)
LPC 0:0/18:1	−0.129	**0.879 (0.780, 0.990)**	−0.082	0.921 (0.782, 1.084)	−0.191	**0.826 (0.701, 0.974)**
LPC 0:0/18:2	−0.209	**0.811 (0.715, 0.920)**	−0.071	0.931 (0.777, 1.116)	−0.307	**0.736 (0.616, 0.879)**
LPC 0:0/20:3	−0.151	**0.860 (0.767, 0.963)**	−0.134	0.875 (0.752, 1.020)	−0.165	0.848 (0.716, 1.004)
LPC 0:0/20:4	−0.161	**0.851 (0.766, 0.945)**	−0.158	**0.854 (0.731, 0.997)**	−0.159	**0.853 (0.739, 0.985)**
LPC 0:0/22:6	−0.194	**0.824 (0.736, 0.922)**	−0.257	**0.773 (0.657, 0.909)**	−0.139	0.870 (0.741, 1.021)
LPC 14:0/0:0	−0.185	**0.831 (0.716, 0.965)**	−0.15	0.861 (0.701, 1.058)	−0.224	**0.799 (0.642, 0.995)**
LPC 15:0/0:0	−0.341	**0.711 (0.640, 0.790)**	−0.322	**0.725 (0.626, 0.840)**	−0.383	**0.682 (0.582, 0.798)**
LPC 16:0/0:0	−0.189	**0.828 (0.743, 0.923)**	−0.19	**0.827 (0.708, 0.967)**	−0.203	**0.816 (0.697, 0.955)**
LPC 16:1/0:0	−0.052	0.949 (0.839, 1.074)	−0.005	0.995 (0.826, 1.197)	−0.067	0.935 (0.793, 1.101)
LPC 17:0/0:0	−0.195	**0.823 (0.736, 0.921)**	−0.194	**0.824 (0.704, 0.965)**	−0.203	**0.816 (0.692, 0.962)**
LPC 18:0/0:0	0.022	1.022 (0.935, 1.117)	0.053	1.054 (0.932, 1.193)	−0.015	0.985 (0.865, 1.121)
LPC 18:1/0:0	−0.202	**0.817 (0.729, 0.916)**	−0.159	0.853 (0.727, 1.002)	−0.256	**0.774 (0.658, 0.910)**
LPC 18:2/0:0	−0.255	**0.775 (0.682, 0.880)**	−0.126	0.882 (0.734, 1.060)	−0.371	**0.69 (0.577, 0.824)**
LPC 18:3/0:0	−0.113	0.893 (0.747, 1.068)	−0.004	0.996 (0.798, 1.244)	−0.241	**0.786 (0.631, 0.980)**
LPC 20:2/0:0	−0.196	**0.822 (0.729, 0.927)**	−0.144	0.866 (0.733, 1.024)	−0.248	**0.780 (0.678, 0.896)**
LPC 20:3/0:0	−0.25	**0.779 (0.689, 0.881)**	−0.211	**0.810 (0.687, 0.955)**	−0.305	**0.737 (0.621, 0.874)**
LPC 20:4/0:0	−0.173	**0.841 (0.754, 0.938)**	−0.137	0.872 (0.746, 1.020)	−0.218	**0.804 (0.692, 0.934)**
LPC 20:5/0:0	−0.268	**0.765 (0.676, 0.866)**	−0.3	**0.741 (0.625, 0.878)**	−0.222	**0.801 (0.678, 0.945)**
LPC 22:5/0:0	−0.243	**0.784 (0.704, 0.873)**	−0.248	**0.780 (0.661, 0.919)**	−0.226	**0.798 (0.695, 0.917)**
LPC 22:6/0:0	−0.223	**0.800 (0.718, 0.892)**	−0.264	**0.768 (0.653, 0.903)**	−0.205	**0.815 (0.702, 0.946)**

Alpha – estimates of the association parameter for the longitudinal variable (metabolite) in the survival sub-model of the joint model (parameter α in Eq. 1); HR – hazard ratios (for a unit increase in transformed metabolite values) computed from the association parameters; 95% CI – 95% confidence intervals for HRs (highlighted in bold are cases where confidence intervals do not contain one). The joint models were estimated using R-package *JM*.

#### Illustrative example: Association of LPC 15:0/0:0 with mortality risk

The strongest association in the total sample in terms of the point estimate was observed for LPC 15:0/0:0 (α = −0.341, HR = 0.711), indicating a 28.9% reduction in the risk of the event (death) happening for each unit increase in (transformed) LPC 15:0/0:0 levels. Similarly, LPC 15:0/0:0 showed the lowest HR among all LPCs in sex-specific analyses (HR = 0.725 in females, HR = 0.682 in males), corresponding to a 27.5% and a 31.8% reduction in the risk of death for each unit increase in LPC 15:0/0:0 in females and males, respectively. This metabolite was selected for additional analyses illustrating different specifications of JM and more detailed investigation of its association with different aging-related characteristics embedded in the structure of SPM; see below. Supplementary Figure 1 displays diagnostic plots assessing the goodness-of-fit and assumptions of JM in applications to LPC 15:0/0:0. Supplementary Figure 1A shows random behavior of standardized marginal residuals around zero (with 94.47% of values lying within the (−1.96, 1.96) interval), validating the assumptions for the within-subjects covariance structure in the longitudinal part of JM. The Cox-Snell residuals plot (Supplementary Figure 1B) also shows the overall good fit of the survival sub-model of JM.

### Applications of JM with shared random effects (JM-SRE)

#### Summary of results

The results of applications of the general JM described in the previous section established the associations of the LPC species with mortality risk. Here, we describe the results of applications of a different type of JM, JM-SRE [34–36] (see Eqs. 3–5 in the section Joint models: General specifications in Materials and Methods), with individual intercepts and slopes of (transformed) LPCs that provide further insight into the relationships between the age-related changes of the metabolites and mortality risk. Table 2 presents the results of applications of the models with individual intercepts (rows with “int” in the column Model, see Eq. 4) and individual intercepts and slopes (rows with “intslope” in the column Model, see Eq. 5) of LPC 15:0/0:0 in the total (rows with “F+M” in the column Sex) and sex-specific samples (rows with “F” and “M” in the column Sex for females and males, respectively).

**Table 2:** Results of applications of joint models with shared random effects (JM-SRE) to measurements of LPC 15:0/0:0 and mortality data in the LLFS: Estimates of the association parameters for the random intercepts and random slopes of the metabolite in the survival sub-model.

**Model**	**Sex**	**Variable**	**α_0_ (α_1_)**	**HR**	**95% CI for HR**	**SD of Variable**
int	F+M	*b_0i_*	**−0.436**	**0.786**	**(0.728, 0.840)**	**0.553**
intslope	F+M	*b_0i_*	**−0.452**	**0.775**	**(0.726, 0.846)**	**0.563**
intslope	F+M	*b_1i_*	−0.765	0.998	(0.994, 1.004)	0.002
int	F	*b_0i_*	**−0.428**	**0.783**	**(0.709, 0.858)**	**0.572**
intslope	F	*b_0i_*	**−0.408**	**0.778**	**(0.694, 0.865)**	**0.615**
intslope	F	*b_1i_*	−0.084	0.999	(0.973, 1.041)	0.011
int	M	*b_0i_*	**−0.461**	**0.785**	**(0.711, 0.854)**	**0.525**
intslope	M	*b_0i_*	**−0.439**	**0.780**	**(0.698, 0.855)**	**0.568**
intslope	M	*b_1i_*	−0.238	0.997	(0.956, 1.006)	0.011

Model – type of joint model (int – random intercept of LPC in survival sub-model, intslope – random intercept and slope of LPC in survival sub-model), see section Joint models: General specifications; Variable – *b*_0*i*_: random intercept of the metabolite, *b*_1*i*_: random slope of the metabolite; α_0_ (α_1_) – estimates of the regression parameters for b_0*i*_ (b_1i_) in respective models; HR – hazard ratios for an increase by a standard deviation of Variable; 95% CI for HR – respective 95% confidence intervals for HRs; SD of Variable – standard deviation of Variable. Highlighted in bold are cases where confidence intervals do not contain one. The JM were estimated using R-package *joineR*. LPC values were transformed (see section Data).

The estimates of the regression parameter *α*_0_ for the random intercept *b*_0*i*_ in the hazard rate (see Eqs. 4–5) are negative (see the values in the column *α*_0_ (*α*_1_) in rows with *b*_0*i*_ in the column Variable), indicating that larger baseline levels of LPC 15:0/0:0 are associated with reduced mortality risks (after adjusting for relevant covariates, see the section Joint models: Specific versions used in applications in Materials and Methods). This observation holds in both models (“int” and “intslope”) and in total and sex-specific analyses. Therefore, similar conclusions about the reduction of the risk of death for increasing baseline LPC 15:0/0:0 levels can be made for the model with individual intercept and slope and for females and males.

Similarly, there are negative estimates of the regression parameter α_1_ for the random slope *b_1i_* in the hazard rate in the random intercept and slope model (see Eq. 5) (see the values in the column α_0_ (α_1_) in rows with *b_1i_* in the column Variable), but the associations were not significant. The negative estimates of this parameter suggest that an increase in the individual slope of LPC 15:0/0:0 might be associated with reduced mortality risk (in the model adjusting for the covariates indicated in the section Joint models: Specific versions used in applications in Materials and Methods). Supplementary Table 1 presents the results of applications of JM-SRE to all 23 LPC species in the total sample. As this table shows, similar associations were observed for many other LPC species, and LPC 15:0/0:0 still has the largest effect size among all LPCs, as in the basic JM analyses presented in the previous section.

#### Illustrative example: Results for the random intercept model in the total sample

The first row in Table 2 presents results for the random intercept (“int”) model applied to the total sample (F+M). It shows that the HR per standard deviation (SD) of an individual intercept in the random intercept model is 0.786. This means a 21.4% reduction in mortality risk for each 0.553 (see the value in the column SD of Variable) increase in transformed LPC 15:0/0:0 levels at the baseline age.

### Applications of SPM

#### Summary of SPM results

In this section, we summarize the results of applications of SPM that further decompose the associations between LPC 15:0/0:0 and mortality risk. These applications consider different aging-related components embedded in the structure of SPM, providing additional details in the context of the aging process. Supplementary Tables 2, 3 present the results of testing of various null hypotheses (H0s) in applications of SPM to measurements of (transformed) LPCs and mortality in the LLFS metabolomics sample. Supplementary Table 4 contains estimates of parameters in the main (unrestricted) model. Supplementary Text Stochastic process models: Interpretation and illustration of components, parameters, and related null hypotheses and Supplementary Figures 2–9 provide detailed descriptions and illustrations of SPM components and parameters, as well as interpretations of related H0s. Below, we provide several illustrative examples describing the results of applications of SPM to LPC 15:0/0:0, referring to rows corresponding to LPC 15:0/0:0 in Supplementary Tables 2–4.

#### Illustrative example: U-shape of mortality as a function of LPC 15:0/0:0

**Figure 1:**
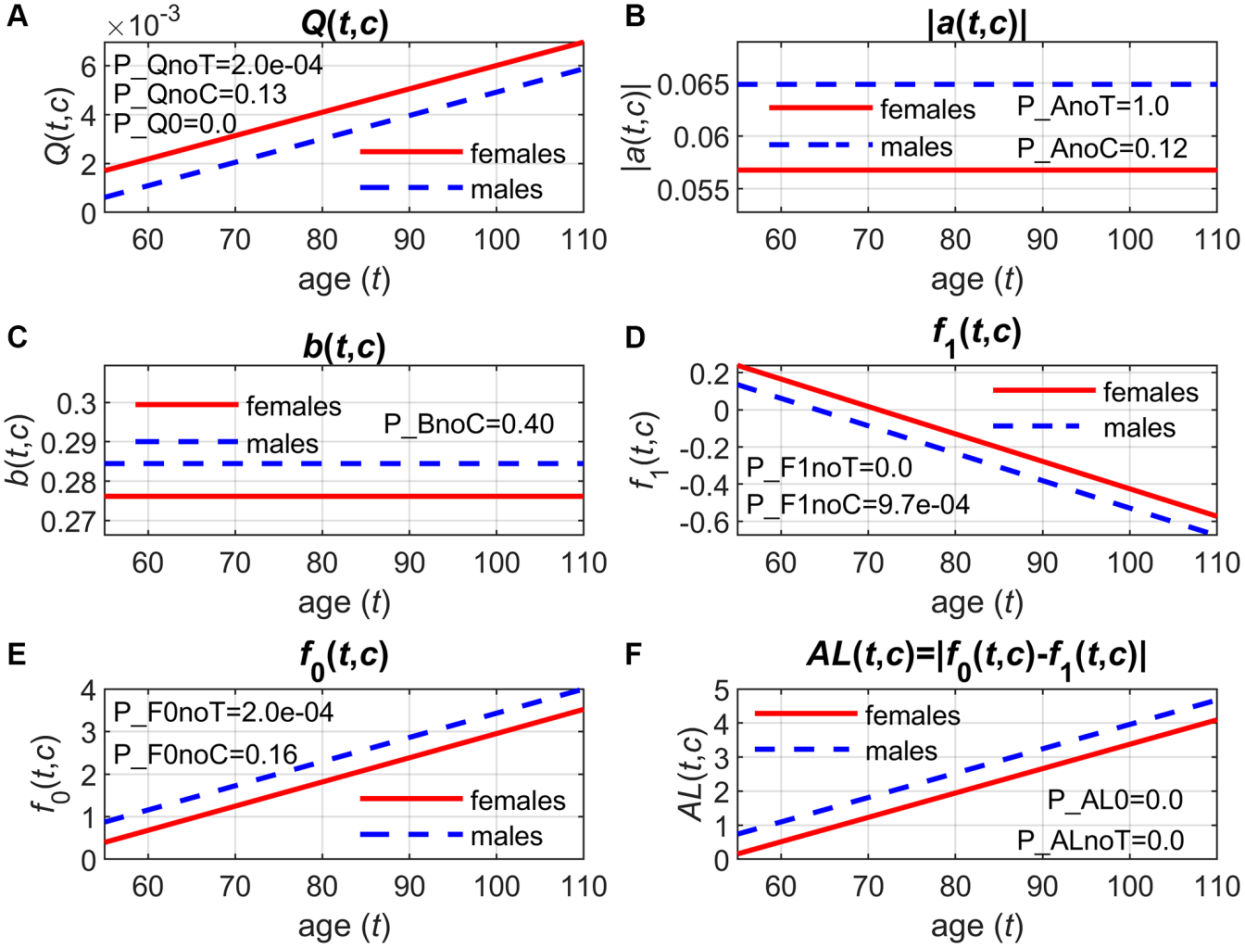
**Applications of stochastic process models to measurements of LPC 15:0/0:0 and mortality data in the LLFS: Estimates of different components of the model.** (**A**) quadratic hazard term (*Q*(*t*, c)); (**B**) adaptive capacity (|*a*(*t*, *c*)|); (**C**) volatility coefficient (*b*(*t*, *c*)); (**D**) equilibrium trajectory (*f*_1_ (*t*, *c*)); (**E**) optimal trajectory (*f*_0_ (*t*, *c*)); (**F**) measure of allostatic load (*AL*(*t*, *c*) = |*f*_0_ (*t*, *c*)–*f*_1_ (*t*, *c*)|); *p*-values shown on the graphs are for different null hypotheses (H0): H0: *Q*(*t*, *c*) = *Q*(*c*) (P_QnoT); H0: *Q*(*t*, *c*) = *Q*(*t*) (P_QnoC); H0: *Q*(*t*, *c*) = 0 (P_Q0); H0: *a*(*t*, *c*) = *a*(*c*) (P_AnoT); H0: *a*(*t*, *c*) = *a*(*t*) (P_AnoC); H0: *b*(*t*, *c*) = *b*(*t*) (P_BnoC); H0: *f*_1_(*t*, *c*) = *f*_1_(*c*) (P_F1noT); H0: *f*_1_(*t*, *c*) = *f*_1_(*t*) (P_F1noC); H0: *f*_0_(*t*, *c*) = *f*_0_(*c*) (P_F0noT); H0: *f*_0_(*t*, *c*) = *f*_0_(*t*) (P_F0noC); H0: *f*_1_(*t*, *c*) = *f*_0_(*t*, *c*), i.e., *AL*(*t*, *c*) = *0* (P_AL0); H0: *f*_1_(*t*, *c*) = *f*_1_(*c*) and *f*_0_(*t*, *c*) = *f*_0_ (c), i.e., *AL*(*t*, *c*) = *AL*(*c*) (P_ALnoT). LPC values were transformed (see Data).

This example illustrates the results presented in columns Qzero and QnoT in Supplementary Table 2, column QnoC in Supplementary Table 3 for LPC 15:0/0:0, and parameters shown in section *Q* (*t*, *c*) in Supplementary Table 4 for this metabolite. The key hypothesis to test before interpreting the SPM results is whether the term in the quadratic part of the hazard (Eq. 7), *Q* (*t*, *c*), is zero. If this were the case, there would be no quadratic part in the hazard, and the age trajectories of LPC 15:0/0:0 would be unrelated to the mortality risk (Supplementary Figure 2B). Rejection of H0: *Q*(*t*, *c*) = 0 (column Qzero in Supplementary Table 2) thus justifies the use of SPM in analyses of LPC 15:0/0:0 and mortality. As Supplementary Figure 10B illustrates, there is a non-zero quadratic part in the hazard, which adds up to the baseline level *μ*_0_ (*t*, *c*) to produce the resulting shape of the total mortality rate as a function of age and LPC 15:0/0:0 shown in Supplementary Figure 10A. Rejection of H0: *Q* (*t*, *c*) = *Q* (*c*) (column QnoT in Supplementary Table 2) and a positive value of the parameter *b*_Q_ (see the respective column in Supplementary Table 4) indicate that the U-shape of the mortality risk as a function of LPC 15:0/0:0 narrows with age (see Supplementary Text, paragraph *“The quadratic hazard term Q* (*t*, *c*)*”* and Supplementary Figure 2). This means that, as individuals grow older, they become more vulnerable to deviations of LPC 15:0/0:0 from the optimal trajectory *f*_0_ (*t*, *c*) because the same deviation of LPC 15:0/0:0 from *f*_0_ (*t*, *c*) results in a larger increase in the mortality risk at older ages compared to younger ages. Figure 1A displays the trajectories of *Q* (*t*, *c*) for females (*c* = 0) and males (*c* = 1), which increase with age as *b_Q_* > 0. As Figure 1A shows, the trajectory of *Q* (*t*, *c*) for males lies below that of females as the respective coefficient shown in column *β_Q_* in Supplementary Table 4 is negative. However, H0: *Q* (*t*, *c*) = *Q* (*t*) (column QnoC in Supplementary Table 3) was not rejected. Therefore, this difference is not statistically significant, so the width of U-shape of the hazard (as a function of LPC 15:0/0:0) does not depend on sex, i.e., there is no sex difference in vulnerability to deviations of trajectories of LPC 15:0/0:0 from the optimal levels *f*_0_ (*t*, *c*).

#### Illustrative example: Feedback and volatility coefficients of LPC 15:0/0:0

This example discusses the results shown in column AnoT in Supplementary Table 2, columns AnoC and BnoC in Supplementary Table 3 for LPC 15:0/0:0, and respective parameters from sections *a* (*t*, *c*) and *b* (*t*, *c*) in Supplementary Table 4. The H0 about the feedback coefficient *a* (*t*, *c*), H0: *a* (*t*, *c*) = *a* (*c*) (column AnoT in Supplementary Table 2), was not rejected. The coefficient *b_Y_* is nearly zero (see the respective column in Supplementary Table 4) so that *a* (*t*, *c*) does not change with age as Figure 1B illustrates. This means that there is no age-related decline in biological resilience related to deviations of trajectories of LPC 15:0/0:0 from the equilibrium levels *f*_1_ (*t*, *c*) (Supplementary Text, paragraph *“The (negative) feedback coefficient a* (*t*, *c*)*”*). The coefficient *β_Y_* is negative (see the respective column in Supplementary Table 4), so the absolute value of *a* (*t*, *c*) is larger in males (Figure 1B); however, this sex difference is not statistically significant as H0: *a* (*t*, *c*) = *a* (*t*) (column AnoC in Supplementary Table 3) was not rejected. In addition, we found that the volatility of LPC 15:0/0:0 was similar in females and males as H0: *b* (*t*, *c*) = *b* (*t*) (column BnoC in Supplementary Table 3) was not rejected. Figure 1C displays the values of the volatility coefficient *b* (*t*, *c*) for females (*c* = 0) and males (*c* = 1), showing a slightly higher volatility of LPC 15:0/0:0 in males (see the positive value of *β_W_* in the respective column in Supplementary Table 4), see also Supplementary Text, paragraph *“The volatility coefficient b* (*t*, *c*)*”*.

#### Illustrative example: Equilibrium trajectories of LPC 15:0/0:0

This example presents the results shown in column F1noT in Supplementary Table 2 and column F1noC in Supplementary Table 3 for LPC 15:0/0:0, and corresponding parameters from section *f*_1_ (*t*, *c*) in Supplementary Table 4. Rejection of H0: *f*_1_ (*t*, *c*) = *f*_1_ (*c*) (column F1noT in Supplementary Table 2) indicates that the equilibrium values of LPC 15:0/0:0 change with age (see also Supplementary Text, paragraph *“The equilibrium trajectory f*_1_ (*t*, *c*)*”*). The estimated value of the slope parameter in *f*_1_ (*t*, *c*) (parameter $b_{f_{1}}$) is negative (see the respective column in Supplementary Table 4), so the equilibrium trajectory of LPC 15:0/0:0 declines with age. Figure 1D shows the trajectories of *f*_1_ (*t*, *c*) for females (*c* = 0) and males (*c* = 1), illustrating the higher equilibrium levels in females (see the negative value of $\beta_{f_{1}}$ in the respective column in Supplementary Table 4 and the rejected H0: *f*_1_ (*t*, *c*) = *f*_1_ (*t*) in column F1noC in Supplementary Table 3).

#### Illustrative example: Optimal trajectories of LPC 15:0/0:0

This example illustrates the results presented in column F0noT in Supplementary Table 2 and column F0noC in Supplementary Table 3 for LPC 15:0/0:0, as well as parameters shown in section *f*_0_ (*t*, *c*) in Supplementary Table 4 for this metabolite. We found that the optimal LPC 15:0/0:0 levels also change with age (H0: *f*_0_ (*t*, *c*) = *f*_0_ (*c*) was rejected, see column F0noT in Supplementary Table 2). This means that the U-shape of the quadratic part in the hazard shifts with age, and so does the minimal mortality level as a function of LPC 15:0/0:0 at specific ages (see also Supplementary Text, paragraph *“The optimal trajectory f*_0_ (*t*, *c*)*”*). The slope of *f*_0_ (*t*, *c*) (parameter $b_{{\text{f}}_{0}}$) is positive (see the respective column in Supplementary Table 4), so the optimal LPC 15:0/0:0 level shifts to larger values with age. Figure 1E illustrates this by displaying the optimal LPC 15:0/0:0 levels *f*_0_ (*t*, *c*) for females (*c* = 0) and males (*c* = 1). Supplementary Figure 10A, 10B present 3D plots of the mortality rate and the quadratic part in the hazard as a function of age and LPC 15:0/0:0, showing the shifting patterns of the U-shape of the quadratic part and the minimal mortality rate (as a function of LPC 15:0/0:0) at specific ages. The estimate of the parameter $\beta_{{\text{f}}_{0}}$ is positive (see the respective column in Supplementary Table 4), which corresponds to higher optimal LPC 15:0/0:0 levels in males (Figure 1E); however, this sex difference is not significant (see column F0noC in Supplementary Table 3).

#### Illustrative example: The gap between equilibrium and optimal trajectories of LPC 15:0/0:0

This example discusses the results shown in columns ALzero and ALnoT in Supplementary Table 2. As Figure 1D, 1E reveal, the equilibrium and optimal trajectories of LPC 15:0/0:0 have opposite directions of change with age (declining vs. increasing), so the absolute value of the difference between these trajectories increases with age (see Figure 1F). This gap between *f*_1_ (*t*, *c*) and *f*_0_ (*t*, *c*), and its increase with age, are significant (see columns ALzero and ALnoT in Supplementary Table 2). This results in an additional mortality risk if LPC 15:0/0:0 is at the equilibrium level, compared to the minimal risk at respective ages given by the baseline mortality *μ*_0_ (*t*, *c*), and this additional mortality “load” increases with age (see also the last paragraph in Supplementary Text).

#### Summary of SPM findings for LPC 15:0/0:0

In brief, we found that:

1. The U-shape of mortality as a function of LPC 15:0/0:0 narrows with age, making older individuals more vulnerable to deviations of LPC 15:0/0:0 concentrations from the trajectory of its optimal values.
2. Equilibrium trajectories of LPC 15:0/0:0 decline with age.
3. Females have higher equilibrium levels of LPC 15:0/0:0 than males.
4. The optimal values of LPC 15:0/0:0 that minimize the mortality risk increase with age.
5. There is a gap between the optimal and equilibrium trajectories, and this gap increases with age.

### Sensitivity analyses

Supplementary Table 5 presents estimates of JM using the familial bootstrap approach [37]. There was one case where the 95% CI for HR in the main calculations (Table 1) did not contain 1.0, but the HR range in the familial bootstrap included 1.0 (highlighted in yellow in Supplementary Table 5). There were three opposite cases (highlighted in grey in Supplementary Table 5). Thus, in most cases, the sensitivity analysis confirmed the results shown in Table 1. In particular, LPC 15:0/0:0 still showed the strongest association with mortality (e.g., HR = 0.713, range: (0.643, 0.778) in the combined females + males analyses).

## DISCUSSION

This work is the first application of two approaches dealing with joint modeling of longitudinal and time-to-event outcomes (JM and SPM) to a large-scale metabolomics study that collected repeated measurements of metabolomics from thousands of participants. These approaches allow for statistically rigorous analyses of repeated measurements of omics data jointly with time-to-event outcomes, avoiding common pitfalls of traditional tools that ignore biological variability/measurement errors in longitudinal outcomes and informative missingness arising due to attrition from mortality (a common situation in aging research) [2, 3, 16]. The basic JM [4] used in our applications allows establishing general associations of longitudinal omics variables with time-to-event outcomes by including the “true” values (i.e., the difference between the observed value and the error term, see Eq. 2) of the variable in the hazard rate and computing respective hazard ratios. The JM-SRE version captures associations between longitudinal and time-to-event outcomes by a latent Gaussian process [34–36], providing different specifications of associations that include individual intercepts and slopes of omics variables in the hazard rate (Eqs. 3–5). These are derived from the random part of the longitudinal sub-model of JM, representing individual characteristics after adjustment for covariates (in the fixed part of the longitudinal sub-model) and the error term. Such models expand analyses by the basic JM and quantify the relations between these individual characteristics of omics trajectories and time-to-event outcomes, e.g., computing hazard ratios for a unit increase in an individual intercept or slope. The SPM digs deeper into the relations between longitudinal changes of omics variables and time-to-event outcomes, decomposing the associations observed in JM into several components representing relevant aging-related characteristics. These characteristics include biological/physiological norms (“sweet spots” [38–40]), allostatic (equilibrium) trajectories and allostatic load, as well as age-related decline in adaptive response to deviations from equilibrium trajectories and age-related increase in vulnerability to deviations from the norms, which represent a decline in biological/physiological robustness and resilience, considered key manifestation of aging [41]. SPM analyses thus can shed more light on relations between age trajectories of omics variables and time-to-event outcomes in the context of aging.

Our applications of JM to data on repeated measurements of different LPC species and mortality in the LLFS found that, for many LPCs, larger levels were associated with reduced mortality risk (or, equivalently, lower levels were associated with increased mortality risk) in the total sample as well as in separate analyses of females and males. This confirms recent results that reported associations of lower LPC levels with unfavorable health outcomes, including mortality (see, e.g., reviews in [25, 32]). For example, in the study of patients with sepsis [42], the non-survival group had significantly lower levels of LPCs 16:0, 17:0, and 18:0 compared to the survival group. In the study involving acute-on-chronic liver failure patients [43], those who died had lower LPC levels than survivors. Decreased LPC levels were significantly associated with increased mortality in bacterial community-acquired pneumonia patients [44]. Reduced LPC levels were associated with poor prognosis (including mortality) in individuals with acute liver failure [45]. All these prior publications reported findings in small samples from specific groups (patients with different diseases/conditions). To the best of our knowledge, our work is the first study that confirmed associations of LPC species with total (all-cause) mortality in a large longitudinal study with thousands of participants and repeated metabolomics measurements.

SPM applications illustrated how the observed associations between the LPC species (taking as an example the variant with the strongest association, LPC 15:0/0:0) and mortality found in JM reflect underlying aging-related characteristics that shape the observed age trajectories of LPCs and their impact on mortality risk. In particular, we found that the U-shape of the mortality risk as a function of LPC 15:0/0:0 narrows with age, reflecting an aging-related decline in robustness to deviations of trajectories of LPC 15:0/0:0 from the optimal levels (that is, those minimizing the mortality risk at a given age). That is, the same magnitude of deviation at older ages leads to a larger increase in the risk than at younger ages. The estimated equilibrium levels of LPC 15:0/0:0 differ by sex, with females having higher (more favorable in terms of the mortality risk) levels than males. The equilibrium levels also decline with age, whereas the optimal levels show an increasing pattern with age. As a result, there is an increasing gap between the optimal and equilibrium levels, which leads to an increased mortality risk with age.

One particular advantage of SPM is that it allows evaluating optimal levels (or ranges [16]) of longitudinal outcomes (e.g., LPC species, as in our applications). Such levels/ranges derived from the model can take into account potential confounders and conceptualize the “optimum” as the levels minimizing the mortality risk (or risks of other events of interest, which, hypothetically, can differ). Such optimal levels do not necessarily coincide with average sex-specific levels for particular ages, as our SPM analyses of LPC 15:0/0:0 illustrate. Thus, SPM applications can expand and complement the ongoing efforts to compute reference values of metabolites for different ages and sexes [46] and can provide additional information that can be used in clinical decision-making processes.

Our SPM results are in line with other studies exploring the LPC-aging nexus. Recently, it was found [31] that higher levels of LPC species were associated with slower biological aging (expressed by two DNA methylation-based metrics). In particular, the LPC species with 15 carbons showed the strongest (negative) association with the biological aging metrics in that study. Lower baseline concentrations and faster declines in levels of several LPC species were associated with a faster decline in skeletal muscle mitochondrial function in longitudinal analyses [47]. Impaired mitochondrial oxidative capacity was previously found to be related to lower levels of several LPC species [48]. Older adults with dual declines in memory and speed showed the most extensive alterations (faster decline) in LPC metabolic profiles [49]. Anti-oxidative stress and anti-inflammatory responses have been suggested as potential biological mechanisms that can explain the observed associations of LPCs with slower biological aging [25, 31, 32]. However, as noted in a recent study [50], LPCs can exhibit opposite signatures, both anti-inflammatory and pro-inflammatory, so that their impact on health can be more ambiguous, with potentially pleiotropic or competing roles that may depend on physiological context, comorbidities, or other factors such as age. As our SPM applications indicate, sex can also be a significant factor contributing to various unobserved aging-related characteristics underlying the LPC trajectories and their relations to mortality. Analyzing sex differences in LPCs (and phospholipids in general) in relation to aging in longitudinal cohort studies is of considerable interest and importance because of the paucity of such studies, and, in particular, considering inconsistencies regarding sex differences in LPC levels during aging observed in prior research [51]. Impacts of other factors on the observed relations between LPC trajectories and mortality can be explored using the tools in this paper, including the genetic underpinnings of the relationships that can be evaluated using relevant tools [14, 52].

We acknowledge that our study has limitations. First, we used simple specifications for the models, e.g., linear functions of age for SPM components. While versatile and flexible, such specifications do not allow exploring more complex non-linear age patterns of respective characteristics. We are limited in our choice by the current availability of repeated measurements of metabolomics in LLFS (up to two per individual). Second, the LLFS is predominantly (>99%) a white sample. Therefore, our findings need confirmation in other studies collecting data for other race/ethnicity groups. Third, we performed analyses of a single metabolite in one-dimensional JM and SPM. While multivariate JM and SPM are available [15, 53], their practical applications in analyses of samples similar in size to this study can be intractable. Relevant dimensionality reduction techniques (e.g., as in our prior works [54, 55]) can be used to mitigate this. Fourth, we used available tools developed for analyses of unrelated samples. Even though sensitivity analyses using the familial bootstrap confirmed the robustness of our results, development and validation of approaches handling relatedness among study participants can benefit future analyses of longitudinal omics data in family-based studies.

## MATERIALS AND METHODS

### Data

The Long Life Family Study (LLFS) [24] is a family-based, longitudinal study of healthy aging and longevity that enrolled participants at four field centers (three in the US: Boston, New York, Pittsburgh, and one in Denmark). The LLFS recruited 4,953 individuals from two-generational families selected for exceptional familial longevity based on the Family Longevity Selection Score [56]. The first in-person evaluation (Visit 1) was conducted in 2006–2009. The second in-person visit (Visit 2) of surviving participants from Visit 1 and newly enrolled participants was completed in 2014–2017. Visit 3 started in 2020 and is ongoing. The participants provided information on socio-demographic indicators, past and current medical conditions, medication use, and physical and cognitive functioning [24]. Annual telephone follow-ups were conducted to collect updates on participants’ vital and health status. All reported deaths were adjudicated by an Adjudication Committee [24]. We used the August 19, 2024 release of the phenotypic LLFS data, with the latest recorded follow-up date on August 7, 2024. Baseline ages were validated using dates of birth from official documents in the US [57] and through the civil registration system in Denmark. Ages at censoring for those alive at the end of the follow-up period were determined from dates of birth and the last follow-up. Ages at death/censoring and an indicator of death were used as time-to-event outcomes in our applications of SPM. In JM applications, time since the baseline was used as the time variable due to the specifics of the JM software used in the analyses.

We used batch 6 (released on October 25, 2023) of LLFS metabolomics data, which provides information on 188 lipid metabolites measured longitudinally in the LLFS participants at Visits 1 and 2. Plasma samples were first processed by using solid-phase extraction kits with both aqueous and organic solvents [58]. Extracted metabolites were then analyzed with liquid chromatography/mass spectrometry (LC/MS). To assess lipid metabolites, reversed-phase chromatography was used in combination with an Agilent 6545 quadrupole time-of-flight mass spectrometer at Washington University in St. Louis. A combination of different tools was used to remove background, annotate adducts, and identify compounds [59–61]. Missing values were imputed using the half-minimum approach (i.e., zeros were replaced by half of the minimum value) [62]. Profiling was performed in batches of approximately 90 samples. Batch correction was accomplished by using a random forest-based batch correction algorithm [63], which outperformed other approaches in the lipid metabolite data [58]. The metabolites were annotated by using standardized names from RefMet, version 07/2023 [64]. We used 23 available lysophosphatidylcholines (LPCs) as the longitudinal outcomes in the analyses described below: LPC 0:0/16:0, LPC 0:0/16:1, LPC 0:0/18:0, LPC 0:0/18:1, LPC 0:0/18:2, LPC 0:0/20:3, LPC 0:0/20:4, LPC 0:0/22:6, LPC 14:0/0:0, LPC 15:0/0:0, LPC 16:0/0:0, LPC 16:1/0:0, LPC 17:0/0:0, LPC 18:0/0:0, LPC 18:1/0:0, LPC 18:2/0:0, LPC 18:3/0:0, LPC 20:2/0:0, LPC 20:3/0:0, LPC 20:4/0:0, LPC 20:5/0:0, LPC 22:5/0:0, LPC 22:6/0:0. Each metabolite was analyzed separately in one-dimensional models. Intensity values were transformed using the inverse-normal transformation [65] (INT) before use in the models.

In total, the LLFS metabolomics sample contains 6,776 measurements of the metabolites (4,221 in Visit 1 and 2,555 in Visit 2). The characteristics of the LLFS metabolomics sample are presented in Supplementary Table 6. Table 3 describes the analytic sample obtained after removal of records with missing information on covariates (see Notes under Supplementary Table 6), which comprised 3,462 participants with 5,066 measurements of each metabolite (3,142 in Visit 1 and 1,924 in Visit 2, with 1,858 participants having one measurement and 1,604 participants with two measurements); 1,245 participants died during the follow-up period.

**Table 3:** Characteristics of the Long Life Family Study metabolomics subsample used in analyses.

**Characteristics**	**Field center**				**Total sample**
	**BU**	**NY**	**PT**	**DK**	
Number of families	218	233	210	77	555
Number of participants at any visit	995	601	862	1,004	3,462
Number of participants at visit 1	947	530	809	856	3,142
Number of participants at visit 2	508	313	448	655	1,924
Number (%) of deceased participants	351 (35.3%)	249 (41.4%)	317 (36.8%)	328 (32.7%)	1,245 (36.0%)
Follow-up period (years) (mean ± SD (range))	10.9 ± 4.7 (0.28, 18.22)	10.6 ± 4.3 (0.72, 18.41)	11.2 ± 4.6 (0.29, 18.50)	12.2 ± 5.1 (0.55, 17.93)	11.3 ± 4.8 (0.28, 18.50)
Age at baseline (mean ± SD (range))	69.7 ± 15.6 (32, 104)	73.1 ± 15.4 (24, 101)	70.4 ± 15.5 (38, 104)	69.2 ± 13.9 (38, 104)	70.3 ± 15.2 (24, 104)
Whites (%)	99.6%	98.8%	99.5%	99.5%	99.4%
Females (%)	55.6%	53.7%	55.3%	55.2%	55.1%
Low educated participants (below high school) (%)	5.8%	6.5%	6.8%	28.2%	12.7%
Smokers (smoked >100 cigarettes in lifetime) (%)	41.7%	45.4%	36.5%	49.1%	43.2%
*APOE* ɛ4 allele carriers (%)	15.3%	19.1%	18.0%	26.9%	20.0%
Medication use: angina (%)	34.7%	31.8%	32.8%	25.7%	31.1%
Medication use: anti-diabetic (%)	6.7%	5.8%	9.0%	6.0%	6.9%
Medication use: anti-hypertensive (%)	52.5%	54.9%	55.1%	47.9%	52.2%
Medication use: lipid-lowering (%)	35.3%	45.3%	41.4%	24.0%	35.3%
No intense physical activity at baseline (%)	70.3%	66.4%	75.3%	75.8%	72.4%
SPPB total score at baseline (mean ± SD (range))	9.9 ± 3.0 (0, 12)	9.6 ± 2.9 (1, 12)	10.0 ± 2.8 (1, 12)	10.1 ± 3.0 (1, 12)	10.0 ± 2.9 (0, 12)
Prevalence of major diseases at baseline (%)	70.5%	71.2%	70.1%	68.9%	70.0%
BMI at baseline (mean ± SD (range))	27.5 ± 5.0 (17, 57)	26.5 ± 4.0 3 (17, 41)	27.7 ± 5.2 (17, 52)	26.4 ± 4.4 (13, 54)	27.1 ± 4.8 (13, 57)

(a) The numbers shown in “Number of deceased participants” and “Follow-up period” correspond to the LLFS data release used in this paper (see Data); (b) “Prevalence of major diseases” includes prevalence of any of the following diseases: heart disease, stroke, lung disease, cancer, hypertension, and diabetes. Abbreviations: BMI: body mass index; BU: Boston; DK: Denmark; NY: New York; PT: Pittsburgh; SD: standard deviation; SPPB: Short Physical Performance Battery.

### Joint models: General specifications

The joint models for longitudinal and time-to-event outcomes (or simply joint models, JM) jointly estimate the parameters of the longitudinal and time-to-event outcomes in the estimation procedure. We used the basic form of JM [4, 5] as implemented in the R-package *JM* [33]. In our applications, JM jointly estimates the parameters of the longitudinal trajectories of LPCs and mortality rates. The JM consists of two sub-models. The survival sub-model of JM represents the mortality rate as a function of a metabolite and other covariates:


$$h_{i}(t\text{​}|\text{​}M_{i}(t),w_{i})=h_{0}(t)\exp\{\gamma^{T}w_{i}+\alpha m_{i}(t)\},\;\;(\text{Eq}\text{. 1})$$

where *h_i_*(*t*|∙) is the mortality rate for the *i*^th^ individual at time point *t*, *m_i_*(*t*) is the “true” (i.e., unobserved) LPC level (see below in the text after Eq. 2) at time *t*, *h*_0_(∙) is the baseline mortality rate, *w_i_* is a vector of baseline covariates, and γ is a vector of respective regression coefficients.

We highlight the main parameter of our interest that we report in respective tables, *α* (a scalar), which is called the association parameter. In JM, the association parameter quantifies the relationship between longitudinal data (repeated measures of (transformed) LPC levels in our case) and time-to-event data (survival times in our application). This parameter is crucial because it helps us understand how changes in the longitudinal measurements are associated with the risk of the respective event. Positive values of *α* indicate that higher values of the longitudinal measurements are associated with an increased risk of the event. Respectively, negative values of *α* imply that higher values of the longitudinal measurements are associated with a decreased risk of the event. A zero association parameter suggests no relationship between the longitudinal measurements and the risk of the event (i.e., changes in the longitudinal measurements do not affect the event’s likelihood). We also report hazard ratios (HRs) computed from the association parameter as *HR* = *exp*(*α*). They are interpreted similarly to HRs from the Cox model, e.g., HR = 1.5 means that for each unit increase in the longitudinal measurement (i.e., (transformed) LPC level), the risk of the event (mortality) increases by 50%, and HR = 0.7 means that for each unit increase in the longitudinal measurement, the risk of the event decreases by 30%.

The longitudinal sub-model of JM in its basic form [4, 5, 33] is a linear mixed effects model that describes changes in the longitudinal outcome ((transformed) LPC levels) as a function of time and other covariates:


$$y_{i}(t)=m_{i}(t)+\varepsilon_{i}(t)=x_{i}^{T}(t)\beta +z_{i}^{T}(t)b_{i}+\varepsilon_{i}(t),\;\;(\text{Eq}\text{. 2})$$

where *y_i_*(*t*) is the observed LPC level at time point *t* in the *i*^th^ individual, *x_i_*(*t*) and *z_i_*(*t*) are the corresponding fixed and random effects, *β* and *b_i_* are the respective vectors of parameters (that model population- and individual-level characteristics of LPC trajectories, respectively), and *ε_i_*(*t*) is the error term (independent of *b_i_*), normally distributed with zero mean and variance *σ*^2^. The difference between the observed value *y_i_*(*t*) and the error term *ε_i_*(*t*), $m_{i}(t)=x_{i}^{T}(t)\beta +z_{i}^{T}(t)b_{i},$ represents the “true” LPC level included in Eq. 1.

In addition to the general form of JM as in Eqs. 1–2, we used the JM versions where the association between the longitudinal and time-to-event outcomes is captured by a latent Gaussian process [34–36], as implemented in the R-package *joineR*. These models (also known as the joint models with shared random effects, JM-SRE) allow for different specifications of associations of individual changes of metabolites with the mortality rate. The general formula for the longitudinal part is as in Eq. 2, but the expression for the hazard rate differs from Eq. 1:


$$h_{i}(t|b_{i},w_{i})=h_{0}(t)\;\text{exp}\{\gamma^{T}w_{i}+D_{i}(t)(\alpha^{T}b_{i})\},\;\;\text{(Eq}\text{. 3)}$$

where *α* is a vector of association parameters corresponding to random effects *b_i_* and *D_i_*(*t*) is the corresponding design matrix. We used two specifications of the JM: the intercept model (“int”) and the intercept and slope model (“intslope”). In the latter case, we used the option “*sepassoc=TRUE*” in function *joint* from the R-package *joineR*. In the “int” model,


$$D_{i}(t)(\alpha^{T}b_{i})=\alpha_{0}b_{0i},\;\;\text{(Eq}\text{. 4)}$$

that is, the individual intercept of LPC (representing individual differences in baseline levels of LPC) enters the hazard rate, and in the “intslope” model,


$$D_{i}(t)(\alpha^{T}b_{i})=\alpha_{0}b_{0i}+\alpha_{1}b_{1}{}_{i}t,\;\;\text{(Eq}\text{. 5)}$$

i.e., the individual intercepts and slopes of LPC (representing individual differences in both baseline levels of LPC and rates of change in LPC levels over time) are both tested for their association with the mortality rate. We report the association parameters *α*_0_ and *α*_1_ in respective tables, which are interpreted similarly to the association parameters in the base JM (see above), for respective variables (individual intercepts and slopes of LPC). We also compute HRs per standard deviation (SD) of the corresponding variables, which are interpreted accordingly (e.g., if *α*_0_ = −0.25 and SD of *b*_0*i*_ is 1.42, then we would have *HR* = *exp* (−0.25 × 1.42) ≈ 0.7, meaning that for each SD increase in the baseline levels of LPC, the mortality risk decreases by about 30%).

### Joint models: Specific versions used in applications

In our applications of the basic form of JM [33], the longitudinal trajectories of different LPC species were modeled by a linear mixed effects model (Eq. 2) with linear random effects, i.e., random intercept and random slope. Time since the baseline visit was used as a time variable (as implemented in the R-package *JM*). Additional covariates were included in the fixed effects part of the longitudinal sub-model of JM: sex (1: male, 0: female), age at baseline visit, country (1: Denmark, 0: USA), education (1: below high school, 0: otherwise), smoking (smoked >100 cigarettes in lifetime: yes (1)/no (0)), medication use (anti-diabetic, lipid-lowering, anti-hypertensive, heart disease) (1: used, 0: not used), APOE4 (1: carriers of apolipoprotein E (*APOE*) ɛ4 allele; 0 – non-carriers of ɛ4). The medications listed above include all available groups constructed by the LLFS investigators from original medication records using the Anatomical Therapeutic Chemical Classification System codes.

The time-to-event outcome (i.e., the mortality rate) was modeled as in Eq. 1, with the following (time-independent) covariates: sex, age at baseline, country, education, smoking, APOE4, physical activity at baseline (1: no intense physical activity at baseline; 0: intense physical activity), Short Physical Performance Battery (SPPB) total score, body mass index (BMI) at baseline, and prevalence of heart disease, stroke, lung disease, cancer, hypertension, or diabetes at baseline (1: prevalence of any of the diseases, 0: no prevalence). In addition, two genetic principal components (PCs) were included as covariates in the hazard rate (we tested models with different numbers of PCs and the results were similar; data not shown). The baseline mortality rate *h*_0_(*t*) was modeled by a piecewise constant function. The pseudo-adaptive Gauss-Hermite quadrature rule [66] was used to approximate the required integrals in the estimation procedure. Analyses were performed in the total sample and separately for females and males (in which case sex was not included as a covariate).

In the specification of JM implemented in the R-package *joineR*, we used the same list of covariates in the longitudinal and survival sub-models. Unlike the *JM* package, the baseline hazard is represented semi-parametrically in *joineR*. Individual values of random intercepts and slopes were used in the expression of the hazard rate as shown in Eqs. 3–5, instead of the “true” level of the metabolite as in Eq. 1.

R version 4.4.1 was used to run the R-packages *JM* (version 1.5-2) and *joineR* (version 1.2.8) estimating respective models.

### Stochastic process models: General specifications

For SPM applications, we used a one-dimensional version with time-dependent components [15]. The age trajectory of a repeatedly measured variable *Y*(*t*,*c*), where *t* is age and *c* denotes covariates, is represented as a stochastic process with the following equation (in our applications, this equation models age trajectories of (transformed) LPC):


$$\text{d}Y(t,c)=a(t,c)\left(Y(t,c)-f_{1}(t,c)\right)dt+b(t,c)\text{d}W(t),\;\;\text{(Eq}\text{. 6)}$$

with initial condition *Y*(*t*_0_, *c*) (*t*_0_ denotes age at entering the study). Note that SPM equations represent individual trajectories/rates; we do not use an index to indicate that *t*, *c*, and *Y*(*t*, *c*) are individual-based quantities, for simplicity of notation and visualization. Here *W*(*t*) is the stochastic (Wiener) process (assumed to be independent of *Y*(*t*_0_, *c*)) that defines random paths of *Y*(*t*, *c*), *b*(*t*, *c*) is the volatility coefficient controlling the volatility of *Y*(*t*, *c*), *f*_1_(*t*, *c*) is the long-term mean of the stochastic process (or the equilibrium trajectory), and *a*(*t*, *c*) is the negative feedback coefficient regulating how fast the trajectory of *Y*(*t*, *c*) returns to *f*_1_(*t*, *c*) when it deviates from it. The SPM expresses the hazard rate (i.e., the mortality rate in our case) as a function of age (*t*), the vector of covariates (*c*), and the value of the longitudinal variable *Y*(*t*, *c*):


$$\mu \left(t,c,Y(t,c)\right)=\mu_{0}(t,c)+Q(t,c){\left(Y(t,c)-f_{0}(t,c)\right)}^{2}.\;\;\text{(Eq}\text{. 7)}$$

Here *μ*_0_(*t*,*c*) is the baseline hazard (i.e., mortality in our case) rate, *Q*(*t*,*c*) is the multiplier scaling the quadratic term of the hazard at different ages and values of covariates, and *f*_0_(*t*,*c*) represents the values of *Y*(*t*,*c*) (i.e., LPC) minimizing the risk (mortality) at age *t* and covariate values *c*.

The main characteristic feature of SPM is that Eqs. 6–7 embed several aging-related concepts (see more details in [16, 17]), thus facilitating more detailed analyses and interpretation of results in the context of aging, compared to analyses by JM (see more details in Supplementary Text Stochastic process models: Interpretation and illustration of components, parameters, and related null hypotheses; Supplementary Figures 2–9 provide further illustration of the model setup and mechanics): (a) *homeostatic regulation*, which is a fundamental feature of a living organism; (b) *allostasis and mean allostatic (“equilibrium”) levels* (*f*_1_(*t*,*c*)), featuring the effect of allostatic adaptation [67], i.e., the LPC levels forced by organism’s regulatory systems functioning at non-optimal levels; (c) *adaptive capacity* (*a*(*t*,*c*)), modeling the rate of adaptive response (associated with *biological resilience* [17, 41, 68]) to any factors causing deviations of *Y*(*t*,*c*) from its dynamic equilibrium levels *f*_1_(*t*,*c*); (d) *physiological or biological optima (“sweet spots”* [38–40]) naturally represented by *f*_0_(*t*,*c*); (e) *vulnerability component of stress resistance* (associated with *biological robustness* [68–71]) captured by the U-(J-)shape of the hazard and regulated by the multiplier *Q*(*t*,*c*) in the quadratic part of the hazard; (f) *allostatic load (AL)* computed as *AL*(*t*,*c*) = |*f*_0_(*t*,*c*) − *f*_1_(*t*,*c*)| and representing the practical realization of the theoretical concept of AL suggested in the literature [67, 72–74] (the larger the value of this AL measure, the greater the price or load is for an organism in terms of an increased mortality risk, compared to the best-case scenario where LPC levels follow the optimal trajectory *f*_0_(*t*,*c*), i.e., when AL = 0).

### Stochastic process models: Specific parameterizations used in applications

For applications, we used the following specification of SPM: (a) the Gompertz baseline hazard (represents a common pattern of mortality rate at adult and old ages): $\text{ln}\;\mu_{0}(t,c)=\ln\;a_{\mu_{0}}+b_{\mu_{0}}(t-t_{\min})+\beta_{\mu }{}_{{}_{0}}\;c;$ (b) constant volatility coefficient (based on our prior simulations showing the best accuracy of parameter estimates for models with constant *b*(.) [15]): *b*(*t*,*c*) = *σ*_1_ + *β*_W_*c*; and (c) linear functions of age for other components (to estimate age trends in the respective components): *a*(*t*,*c*) = *a*_Y_ + *b*_Y_(*t* − *t*_min_) + β_Y_*c*, where *a*_Y_ < 0, *b*_Y_ ≥ 0 and *t*_min_ = 50; $f_{o}(t,c)=a_{f_{0}}+b_{f_{0}}(t-t_{\min})+\beta_{f_{0}}c$; $f_{1}(t,c)=a_{f_{1}}+b_{f_{1}}(t-t_{\min})+\beta_{f_{1}}c$; $Q\;\;(t,c)=a_{Q}+b_{Q}(t-t_{\min})+\beta_{Q}c;$ and $Y(t_{0},c)∼\;N(f_{1}(t_{0},c),\sigma_{0}^{2}).$ Based on our prior experience (dictated by technical complexities of the estimation algorithm), we included all covariates used in JM (except age at baseline because age is used as the time variable in SPM) in *μ*_0_(*t*,*c*), whereas only one covariate (sex) was included in all other components.

In-house MATLAB codes (run in MATLAB version R2024a) implementing estimation algorithms with covariates in discrete-time approximations of SPM [18, 55] were used to estimate SPM parameters. Likelihood ratio tests were used to test several null hypotheses (H0s) about the functional forms of each model’s components (i.e., to test whether they depend on age and on respective covariates, or are non-zero). First, an “unrestricted” model (with the parameterization presented above) with no restrictions on parameters was estimated. Then, other models that contain one or more restrictions on parameters were estimated to test respective H0s:

1. H0: *Q*(*t*,*c*) (Qzero; interpretation: no quadratic term in the hazard);
2. H0: *Q*(*t*,*c*) = *Q*(*c*), i.e., *b*_Q_ = 0 (QnoT; the term in the quadratic part of the hazard does not depend on age, i.e., robustness to deviations of LPC from the optimal trajectory does not depend on age);
3. H0: *Q*(*t*,*c*) = *Q*(*t*), i.e., *β*_Q_ = 0 (QnoC; the term in the quadratic part of the hazard does not depend on sex, i.e., robustness to deviations of LPC from the optimal trajectory does not depend on sex);
4. H0: *a*(*t*,*c*) = *a*(*c*), i.e., *b*_Y_ = 0 (AnoT; the feedback coefficient does not depend on age, i.e., the adaptive capacity (resilience) is age-independent);
5. H0: *a*(*t*,*c*) = *a*(*t*), i.e., *β*_Y_ = 0 (AnoC; the feedback coefficient does not depend on sex, i.e., the adaptive capacity (resilience) is the same in females and males);
6. H0: *b*(*t*,*c*) = *b*(*t*), i.e., *β*_W_ = 0 (BnoC; the volatility coefficient does not depend on sex);
7. H0: *f*_1_(*t*,*c*) = *f*_1_(*c*), i.e., $b_{{\text{f}}_{1}}=0$ (F1noT; equilibrium LPC levels are the same for all ages);
8. H0: *f*_1_(*t*,*c*) = *f*_1_(*t*), i.e., $\beta_{{\text{f}}_{1}}=0$ (F1noC; equilibrium LPC levels do not differ by sex);
9. H0: *f*_0_(*t*,*c*) = *f*_0_(*c*), i.e., $b_{{\text{f}}_{0}}=0$ (F0noT; LPC “sweet spots” are the same for all ages);
10. H0: *f*_0_(*t*,*c*) = *f*_0_(*t*), i.e., $\beta_{{\text{f}}_{0}}=0$ (F0noC; optimal LPC levels minimizing mortality risk coincide for females and males);
11. H0: *f*_1_(*t*,*c*) = *f*_0_(*t,c*), i.e., *AL*(*t*,*c*) = 0 (ALzero, zero AL);
12. H0: *f*_1_(*t*,*c*) = *f*_1_(*c*) and *f*_0_(*t*,*c*) = *f*_0_(*c*), i.e., $\beta_{{\text{f}}_{1}}=0$ and $b_{{\text{f}}_{0}}=0$, or *AL*(*t*, *c*) = *AL*(*c*) (ALnoT, AL does not accumulate with age).

### Sensitivity analyses

The LLFS is a family-based study that contains related individuals. Currently available JM tools allowing analyses of related individuals (R-packages *merlin* and *rstanarm*) were not usable for our applications because of technical issues. SPM tools for related samples currently do not exist. Therefore, we used the available tools for unrelated individuals. To test whether this could affect our results, we performed sensitivity analyses implementing the “familial bootstrap” approach [37]. Specifically, we collected estimates of the JM from 100 bootstrap samples constructed from data on the families generated (with replacement) from the original analytic sample (note that, even though the number of families in each generated sample was the same, the numbers of individuals were different). Then, we computed relevant quantities from all 100 samples (e.g., medians of hazard ratios of the association parameter *α* (Eq. 1) in JM, along with the range of the hazard ratios). The respective estimates are provided in Supplementary Materials and discussed in Results.

## Supplementary Materials

Supplementary Material

Supplementary Figures

Supplementary Tables
