# Supplementary material for: Methods for joint modeling of longitudinal omics data and time-to-event outcomes: applications to lysophosphatidylcholines in connection to aging and mortality in the Long Life Family Study
Source: Aging (Albany NY). 2025 May 27;17(5):1221–60. doi: 10.18632/aging.206259 (PMC12151508; doi:10.18632/aging.206259)
Supplement: Supplementary Material [file aging-17-206259-s001.pdf]

SUPPLEMENTARY MATERIALS

Stochastic process models: Interpretation and illustration of components, parameters, and related null hypotheses

As described in the main text (section Stochastic process models: General specifications), for stochastic process models (SPM) applications, we used a one-dimensional version with time-dependent components [1]. This model has two equations: one for modeling individual age trajectories of LPC species $Y(t,c)$ (where t is age and c denotes covariates; see section Stochastic process models: Specific parameterizations used in applications in the main text) and one representing individual mortality rate $\mu\big(t,c,Y(t,c)\big)$ as a function of age, covariates, and LPC levels:

$$dY(t,c) = a(t,c)\big(Y(t,c) - f_1(t,c)\big)dt + b(t,c)dW(t), \quad \text{(Eq. 6)}$$

$$\mu\big(t,c,Y(t,c)\big) = \mu_0(t,c) + Q(t,c)\big(Y(t,c) - f_0(t,c)\big)^2. \quad \text{(Eq. 7)}$$

Note that these equations model individual trajectories/rates; we do not use an index to indicate that t, c, and $Y(t,c)$ are individual-based quantities, for simplicity of notation and visualization. Here, $Y(t_0,c)$ is the initial condition (t_0 denotes age at entering the study) and $W(t)$ is the stochastic Wiener process (also known as Brownian motion [2]), which is assumed to be independent of $Y(t_0,\mathrm{c})$. This process defines random paths of $Y(t,c)$. Below, we provide a detailed description of the model's components and parameters, and illustrate the meaning of related null hypotheses. This can assist in the interpretation of SPM results presented in Figure 1 and Supplementary Figure 10A, 10B, and Supplementary Tables 2–4.

Baseline mortality rate $\mu_0(t,c)$ represents the part of the mortality rate in Eq. (7) that is not related to LPC ($Y(t,c)$). As SPM is a parametric model, we need to specify the parametric form of $\mu_0(t,c)$, along with other components. For our applications, we chose the Gompertz baseline hazard, which is commonly used in demography to represent a typical pattern of mortality rate at adult and old ages. In this parameterization, the logarithm of the baseline mortality rate is a linear function of age and covariates: $\ln \mu_0(t,c) = \ln a_{\mu_0} + b_{\mu_0}(t - t_{\min}) + \beta_{\mu_0} c$, where $t_{\min} = 50$ in our applications. Here, a_{μ_0} is the baseline mortality rate corresponding to age $t_{\min}$ and zero values of covariates in a (column) vector c. Parameter b_{μ_0} represents the rate of change in $\ln \mu_0(t,c)$ with age, and β_{μ_0} is a (row) vector of parameters corresponding to covariates in c. The baseline mortality rate is not of direct interest in our applications. Therefore, we do not test any null hypothesis related to $\mu_0(t,c)$.

The quadratic hazard term $Q(t,c)$ (assumed to be non-negative for all t and c) is the multiplier scaling the quadratic component of the hazard in Eq. (7). Such a quadratic shape of the mortality rate used in SPM reflects common epidemiological observations (including our own research [3–6]) that mortality as a function of various biomarkers has a U-shape. This means that there is an optimal value of a biomarker ($f_0(t,c)$) that minimizes the mortality risk at a specific age (and specific values of covariates, if relevant) and that the deviations of the biomarker to smaller or larger values induce an additional mortality risk. This is captured by the quadratic shape of the mortality rate, and $Q(t,c)$ controls the width of the U-shape. We test several null hypotheses (H0) related to this component of SPM.

First, we test H0: $Q(t,c) = 0$ (denoted Qzero). This is the key H0 to test because if we are not able to reject it, it indicates that the respective biomarker (LPC) is not related to the risk of death, making testing any other H0s for that particular LPC irrelevant. Supplementary Figure 2A illustrates the situation when Qzero is rejected, i.e., LPC values are related to the mortality risk (as illustrated by a U-shape of the mortality rate). Supplementary Figure 2B, conversely, shows the case when $Q(t,c) = 0$, so that the mortality rate equals $\mu_0(t,c)$ and it is independent of LPC values.

Second, we test H0 about the age pattern of $Q(t,c)$. We use a parsimonious parameterization of $Q(t,c)$: $Q(t,c) = a_Q + b_Q(t - t_{\min}) + \beta_Q c$. The parameter a_Q corresponds to the "baseline" width of the U-shape at age $t_{\min}$ and zero covariate(s) c. We use only one covariate (sex) in $Q(t,c)$, so this corresponds to the width of the U-shape in 50-year-old females. The parameter b_Q models how the width of the U-shape changes with age. We test H0: $b_Q = 0$ (QnoT). Supplementary Figure 3A presents three possible age patterns of $Q(t,c)$: age independent ($b_Q = 0$), declining with age ($b_Q < 0$), and increasing with age ($b_Q > 0$). Supplementary Figure 3B–3D display corresponding values of the quadratic part in the hazard (i.e., $Q(t,c)\big(Y(t,c) - f_0(t,c)\big)^2$) for different ages and LPC levels (with c = 0 for the simplicity of illustration). For example, if $b_Q > 0$ (Supplementary Figure 3D), then the U-shape of the mortality rate shrinks with age so that the same deviation of LPC from an optimal (age-specific) level results in a larger additional mortality

risk at older ages compared to younger ages, i.e., the impact of deviations of LPC trajectories from optimal levels aggravates with age. Such observations would correspond to an increase in vulnerability to deviations of LPC from optimal values, which is the manifestation of *age-related decline in biological robustness* [7, 8].

Third, we test H0 about the dependence of $Q(t,c)$ on c (sex-dependence), H0: $\beta_Q = 0$ (QnoC). The parameter β_Q specifies how/if the U-shape of the mortality rate as a function of LPC is different in females and males. If $\beta_Q = 0$, then the width of the U-shape is the same in females and males at each age. If $\beta_Q < 0$ ($\beta_Q > 0$), then the U-shape is wider (narrower) in males compared to females at each age. Supplementary Figure 4A, 4B show the patterns of $Q(t,c)$ and the corresponding values of the quadratic part in the hazard ($Q(t,c)\big(Y(t,c) - f_0(t,c)\big)^2$) for females and males when $\beta_Q > 0$ (assuming zero optimal levels $f_0(t,c)$ in females and males, for the simplicity of illustration). In this case, the U-shape is narrower in males at each age, and the same deviation of LPC from the optimal level results in higher additional mortality risk in males compared to females (thus increasing the overall mortality risk in males even if the baseline mortality rates are the same in both sexes).

The (negative) feedback coefficient $a(t,c)$ in Eq. (6) regulates the dynamic behavior of LPC trajectories ($Y(t,c)$). The particular form of the equation used for modeling $Y(t,c)$ in SPM was selected to incorporate *homeostatic regulation* in the model, which is a fundamental feature of living organisms. The trajectory of $Y(t,c)$ modeled by this equation tends to move back to its long-term mean trajectory (or "equilibrium" level) ($f_1(t,c)$) if it deviates from this equilibrium trajectory. For this to happen, the feedback coefficient has to be negative, hence the restriction used in the model: $a(t,c) < 0$, for all values of age t and covariate(s) c observed in the data. The rate of return to the equilibrium level is regulated by this coefficient $a(t,c)$. The larger the absolute value of the feedback coefficient $a(t,c)$, the faster $Y(t,c)$ returns to its equilibrium level. This coefficient is also called the *adaptive capacity* [1] because it represents the rate of adaptive response (associated with *biological resilience* [7, 9, 10]) to any factors causing deviations of $Y(t,c)$ from their dynamic equilibrium levels $f_1(t,c)$.

In our applications, we use a linear function for $a(t,c)$: $a(t,c) = a_Y + b_Y(t - t_{\min}) + \beta_Y c$, where $a_Y < 0$, $b_Y \geq 0$. The parameter a_Y represents the "baseline" value of this coefficient corresponding to age $t_{\min}$ and zero covariate(s) c (i.e., 50 years old females in our case). The parameter b_Y models the rate of change in $a(t,c)$ with age. We test H0: $b_Y = 0$ (AnoT). Supplementary Figure 5A shows examples of the absolute value of the feedback coefficient ($|a(t,c)|$) for zero and positive b_Y (with $c = 0$ for the simplicity of illustration). Supplementary Figure 5B displays sample trajectories of $Y(t,c)$ in these two cases. As one can see, in case of a positive b_Y (when the absolute value of the feedback coefficient becomes smaller with age), it takes more time for a trajectory of $Y(t,c)$ to go back to the equilibrium level $f_1(t,c)$ at older ages compared to younger ages. This illustrates the *aging-related decline in adaptive capacity* or the associated notion of the *decline in biological resilience*, which is a key manifestation of aging [9]. Note that we show the absolute value of $a(t,c)$, $|a(t,c)|$, rather than $a(t,c)$ in Supplementary Figure 5 so that a decline in the displayed quantity would have the interpretation of a decline in adaptive capacity/biological resilience.

We also test H0: $\beta_Y = 0$ (AnoC). The parameter β_Y specifies the difference in the baseline levels of the adaptive capacity between males and females. If it is zero, then the baseline level of $a(t,c)$ is the same in females and males. If $\beta_Y > 0$ ($\beta_Y < 0$), then females are more (less) resilient compared to males in terms of a faster (slower) rate of return of $Y(t,c)$ (LPC) to its equilibrium levels. Supplementary Figure 5C, 5D present examples of $|a(t,c)|$ and sample trajectories of $Y(t,c)$ in females and males. This illustrates the situation when $\beta_Y > 0$ corresponding to better adaptive capacity in females (i.e., a faster return of $Y(t,c)$ to the equilibrium level $f_1(t,c)$).

The volatility coefficient $b(t,c)$ controls the volatility of the process $Y(t,c)$. The volatility of $Y(t,c)$ represents the intensity of the random fluctuations (or noise) in the process. It determines how much the process can deviate from its mean due to random impacts. Higher volatility means larger deviations from the mean, while lower volatility indicates smaller deviations. Supplementary Figure 6 shows examples of the process $Y(t,c)$ with higher and lower volatility. Based on our prior simulations showing the best accuracy of parameter estimates for models with a constant volatility coefficient [1], we use the following specification of $b(t,c)$: $b(t,c) = \sigma_1 + \beta_W c$ (with the constraint: $b(t,c) > 0$ for all values of covariates c, i.e., sex in our case). In our applications, σ_1 represents the value of this coefficient in females and $\sigma_1 + \beta_W$ is the volatility coefficient in males, which can be larger ($\beta_W > 0$), smaller ($\beta_W < 0$), or the same ($\beta_W = 0$) as in females. We test the respective H0: $\beta_W = 0$ (BnoC) to determine if the volatility of LPC is sex-specific.

The equilibrium trajectory $f_1(t,c)$ represents the long-term mean of the process $Y(t,c)$ (see the paragraph

describing $a(t,c)$). This SPM component is also known as the "*mean allostatic trajectory*" because it features the effect of allostatic adaptation [11], i.e., the LPC levels forced by an organism's regulatory systems functioning in non-optimal conditions (as postulated by the theory of allostasis [11–13]). In our applications, we assume that the equilibrium LPC levels can depend on age (t) and covariates (c) (i.e., sex). We use the following specification of $f_1(t,c)$: $f_1(t,c) = a_{f_1} + b_{f_1}(t - t_{\min}) + \beta_{f_1}c$. Here, the parameter a_{f_1} is the equilibrium LPC level at age $t_{\min}$ and zero values of covariates c (i.e., in 50-year-old females). The parameter b_{f_1} quantifies the rate of change in the equilibrium LPC level with age, which can increase ($b_{f_1} > 0$), decrease ($b_{f_1} < 0$), or be stable ($b_{f_1} = 0$). The parameter β_{f_1} determines how/if the baseline equilibrium LPC level differs by sex: higher ($\beta_{f_1} > 0$) or lower ($\beta_{f_1} < 0$) in males, or sex-independent ($\beta_{f_1} = 0$). We test two null hypotheses about $f_1(t,c)$ to determine its age pattern and dependence on sex: H0: $b_{f_1} = 0$ (F1noT), i.e., equilibrium LPC levels are the same for all ages, and H0: $\beta_{f_1} = 0$ (F1noC), i.e., the baseline equilibrium LPC levels do not differ by sex. Supplementary Figure 7A, 7B display sample trajectories of $Y(t,c)$ for different age (Supplementary Figure 7A) and sex (Supplementary Figure 7B) patterns of $f_1(t,c)$.

The optimal trajectory $f_0(t,c)$ represents the values of $Y(t,c)$ minimizing the risk at age t and covariate values c. It is interpreted as a *physiological or biological optimum* (also known as *"sweet spots"* [14–16]). In our applications, $f_0(t,c)$ models LPC levels minimizing mortality risks at respective ages and covariate values. We parameterize $f_0(t,c)$ as a linear function: $f_0(t,c) = a_{f_0} + b_{f_0}(t - t_{\min}) + \beta_{f_0}c$. Here, a_{f_0} is the LPC value corresponding to the minimal mortality risk at age $t_{\min}$ for individuals with zero c (i.e., 50 years old females). The parameter b_{f_0} defines the rate of change in the optimal LPC level with age. It can increase ($b_{f_0} > 0$), decrease ($b_{f_0} < 0$), or remain stable ($b_{f_0} = 0$) with age. The parameter β_{f_0} specifies how/if the baseline optimal LPC level differs by sex: higher ($\beta_{f_0} > 0$) or lower ($\beta_{f_0} < 0$) in males, or sex-independent ($\beta_{f_0} = 0$). Similar to $f_1(t,c)$, we test two null hypotheses about $f_0(t,c)$ to determine its age pattern and dependence on sex: H0: $b_{f_0} = 0$ (F0noT), i.e., optimal LPC levels do not change with age, and H0: $\beta_{f_0} = 0$ (F0noC), i.e., the baseline optimal LPC levels are equal in females and males. Supplementary Figure 8A, 8B present the quadratic part in the hazard (i.e., $Q(t,c)\big(Y(t,c) - f_0(t,c)\big)^2$) for different ages and LPC levels (with c = 0 for the simplicity of illustration) in the case of increasing and declining optimal levels. Supplementary Figure 8C, 8D show corresponding mortality rates $\mu\big(t,c,Y(t,c)\big)$ for different ages and LPC levels (with zero covariates c, for the purpose of this illustration). We also assumed in this illustration that $Q(t,c)$ does not depend on age t so that the width of the U-shape of mortality as a function of the biomarker $Y(t,c)$ is the same for all ages. As Supplementary Figure 8A shows, if the optimal trajectory increases with age, then the U-shape of the quadratic part in the hazard shifts to the right (to larger values of $Y(t,c)$) so that smaller values of $Y(t,c)$ result in a larger additional risk compared to the baseline mortality $\mu_0(t,c)$ observed at the optimal level $f_0(t,c)$ (see Supplementary Figure 8C). Conversely, when the optimal trajectory declines with age (Supplementary Figure 8B), the parabola in the hazard rate shifts to the left (to smaller values of $Y(t,c)$) so that larger values of $Y(t,c)$ induce a larger additional risk (Supplementary Figure 8D).

Note that the equilibrium and optimal trajectories can be different, and the absolute value of this difference, $AL(t,c) = |f_0(t,c) - f_1(t,c)|$, is related to the practical realization of the theoretical concept of the *allostatic load (AL)* suggested in the literature [11–13, 17]. If the optimal and equilibrium trajectories coincide (i.e., $AL(t,c) = 0$), then LPC trajectories ($Y(t,c)$) tend to converge to $f_0(t,c)$ so that the mortality rate $\mu\big(t,c,Y(t,c)\big)$ gets closer to the baseline level $\mu_0(t,c)$ as the quadratic part in Eq. (7) (i.e., $Q(t,c)\big(Y(t,c) - f_0(t,c)\big)^2$) gets close to zero. However, if the equilibrium trajectory differs from the optimal one, then LPC values tend to a trajectory which is different from that minimizing the mortality rate. As a result, the mortality rate fluctuates around the level $\mu_0(t,c) + Q(t,c)\big(f_1(t,c) - f_0(t,c)\big)^2$. As $Q(t,c) \geq 0$ by the assumption of SPM, this means that this level is higher than the baseline mortality $\mu_0(t,c)$. The larger the value of this measure $AL(t,c)$, the larger this additional mortality risk ("load") $Q(t,c)\big(f_1(t,c) - f_0(t,c)\big)^2$. We test two H0s related to $AL(t,c)$. First, we test H0: $f_1(t,c) = f_0(t,c)$, i.e., $AL(t,c) = 0$ (ALzero), that is, the optimal and equilibrium trajectories are the same. Second, we test H0: $b_{f_1} = 0$ and $b_{f_0} = 0$ (ALnoT), i.e., that the difference between the optimal and equilibrium trajectories does not change with age. Supplementary Figure 9A, 9B illustrate the quadratic part in the hazard ($Q(t,c)\big(f_1(t,c) - f_0(t,c)\big)^2$) and the mortality rate ($\mu\big(t,c,Y(t,c)\big)$) (Eq. 7) evaluated at the equilibrium $f_1(t,c)$ and optimal ($f_0(t,c)$) levels for different ages t (with c = 0 for the simplicity of illustration). This shows the case when the optimal and equilibrium trajectories diverge at older ages, i.e., when $AL(t,c)$ increases with

age. As this illustrative example shows, if the equilibrium and optimal levels coincide at age 50, then there is no additional mortality risk when LPC is at the equilibrium level. Therefore, the mortality rate for an “average” individual with LPC at the equilibrium level equals the optimal (baseline) level $\mu_0(50, c)$. However, if the equilibrium and optimal trajectories diverge with age, then the mortality rate of a centenarian whose LPC level follows the equilibrium trajectory will be about 0.32 higher than the mortality rate of a centenarian with the optimal LPC level for that age.

SUPPLEMENTARY REFERENCES

1. Yashin AI, Arbeev KG, Akushevich I, Kulminski A, Ukraintseva SV, Stallard E, Land KC. The quadratic hazard model for analyzing longitudinal data on aging, health, and the life span. Phys Life Rev. 2012; 9:177–88. https://doi.org/10.1016/j.plrev.2012.05.002 PMID:22633776

2. Durrett R. Brownian Motion: Cambridge University Press). 2019.

3. Arbeev KG, Cohen AA, Arbeeva LS, Milot E, Stallard E, Kulminski AM, Akushevich I, Ukraintseva SV, Christensen K, Yashin AI. Optimal Versus Realized Trajectories of Physiological Dysregulation in Aging and Their Relation to Sex-Specific Mortality Risk. Front Public Health. 2016; 4:3. https://doi.org/10.3389/fpubh.2016.00003 PMID:26835445

4. Yashin AI, Arbeev KG, Akushevich I, Ukraintseva SV, Kulminski A, Arbeeva LS, Culminskaya I. Exceptional survivors have lower age trajectories of blood glucose: lessons from longitudinal data. Biogerontology. 2010; 11:257–65. https://doi.org/10.1007/s10522-009-9243-1 PMID:19644762

5. Yashin AI, Arbeev KG, Ukraintseva SV, Akushevich I and Kulminski A. Patterns of aging related changes on the way to 100: An approach to studying aging, mortality, and longevity from longitudinal data. N Amer Actuarial J. 2012; 16:403–33.

6. Yashin AI, Ukraintseva SV, Arbeev KG, Akushevich I, Arbeeva LS, Kulminski AM. Maintaining physiological state for exceptional survival: What is the normal level of blood glucose and does it change with age? Mech Ageing Dev. 2009; 130:611–8. https://doi.org/10.1016/j.mad.2009.07.004 PMID:19635493

7. Ukraintseva S, Yashin AI, Arbeev KG. Resilience Versus Robustness in Aging. J Gerontol A Biol Sci Med Sci. 2016; 71:1533–4. https://doi.org/10.1093/gerona/glw083 PMID:27146372

8. Arbeev KG, Ukraintseva SV, Bagley O, Zhbannikov IY, Cohen AA, Kulminski AM, Yashin AI. "Physiological Dysregulation" as a Promising Measure of Robustness and Resilience in Studies of Aging and a New Indicator of Preclinical Disease. J Gerontol A Biol Sci Med Sci. 2019; 74:462–8. https://doi.org/10.1093/gerona/gly136 PMID:29939206

9. Ukraintseva S, Arbeev K, Duan M, Akushevich I, Kulminski A, Stallard E, Yashin A. Decline in biological resilience as key manifestation of aging: Potential mechanisms and role in health and longevity. Mech Ageing Dev. 2021; 194:111418. https://doi.org/10.1016/j.mad.2020.111418 PMID:33340523

10. Arbeev KG, Bagley O, Yashkin AP, Duan H, Akushevich I, Ukraintseva SV, Yashin AI. Understanding Alzheimer's disease in the context of aging: Findings from applications of stochastic process models to the Health and Retirement Study. Mech Ageing Dev. 2023; 211:111791. https://doi.org/10.1016/j.mad.2023.111791 PMID:36796730

11. McEwen BS, Wingfield JC. The concept of allostasis in biology and biomedicine. Horm Behav. 2003; 43:2–15. https://doi.org/10.1016/s0018-506x(02)00024-7 PMID:12614627

12. Sterling P and Eyer J. Allostasis: A New Paradigm to Explain Arousal Pathology. In: Fisher S and Reason J, eds. Handbook of Life Stress, Cognition and Health. (New York: John Wiley & Sons). 1988. 629–49.

13. McEwen BS. Stress, adaptation, and disease. Allostasis and allostatic load. Ann N Y Acad Sci. 1998; 840:33–44. https://doi.org/10.1111/j.1749-6632.1998.tb09546.x PMID:9629234

14. Johnson JD, Alejandro EU. Control of pancreatic beta-cell fate by insulin signaling: The sweet spot hypothesis. Cell Cycle. 2008; 7:1343–7. https://doi.org/10.4161/cc.7.10.5865 PMID:18418065

15. Lee JH, Han K, Huh JH. The sweet spot: fasting glucose, cardiovascular disease, and mortality in older adults with diabetes: a nationwide population-based study. Cardiovasc Diabetol. 2020; 19:44. https://doi.org/10.1186/s12933-020-01021-8 PMID:32238157

16. Vishnyakova O, Song X, Rockwood K, Elliott LT, Brooks-Wilson A. Physiological phenotypes have

optimal values relevant to healthy aging: sweet spots deduced from the Canadian Longitudinal Study on Aging. Geroscience. 2024; 46:1589–605. https://doi.org/10.1007/s11357-023-00895-2 PMID:37688655

17. McEwen BS, Stellar E. Stress and the individual. Mechanisms leading to disease. Arch Intern Med. 1993; 153:2093–101. PMID:8379800

18. Borecki IB, Province MA. Genetic and genomic discovery using family studies. Circulation. 2008; 118:1057–63. https://doi.org/10.1161/CIRCULATIONAHA.107.714592 PMID:18765388

Code implementing likelihood estimation procedure of SPM

This section describes the code that can be used to estimate the likelihood function of the Stochastic Process Model (SPM) used in the paper. It provides the code for "unrestricted" model, which can be modified to specify one or more restrictions on parameters to perform hypothesis testing presented in the text.

The function estimates the likelihood for SPM represented by equations:

$$dY(t,c) = a(t,c)(Y(t,c) - f_1(t,c))dt + b(t,c)dW(t)$$

$$\mu(t,c,Y(t,c)) = \mu_0(t,c) + Q(t,c)(Y(t,c) - f_0(t,c))^2$$

with the following specification of components:

$$a(t,c) = a_Y + b_Y(t - t_{min}) + \beta_Y c, a_Y < 0, b_Y \geq 0,$$

$$f_1(t,c) = a_{f_1} + b_{f_1}(t - t_{min}) + \beta_{f_1} c,$$

$$b(t,c) = \sigma_1 + \beta_W c,$$

$$\ln \mu_0(t,c) = \ln a_{\mu_0} + b_{\mu_0}(t - t_{min}) + \beta_{\mu_0} c,$$

$$Q(t,c) = a_Q + b_Q(t - t_{min}) + \beta_Q c,$$

$$f_0(t,c) = a_{f_0} + b_{f_0}(t - t_{min}) + \beta_{f_0} c,$$

$$Y(t_0,c) \sim N(f_1(t_0,c), \sigma_0).$$

Syntax:

```
function lnLik = LogLik(Param, DataSPM, t_min, NamesCovar)
```

Parameters:

Param - a column vector of model parameters in the following order:

$$\ln a_{\mu_0}, b_{\mu_0}, \beta_{\mu_0}, a_Q, b_Q, \beta_Q, a_Y, b_Y, \beta_Y, \sigma_0, \sigma_1, \beta_W, a_{f_1}, b_{f_1}, \beta_{f_1}, a_{f_0}, b_{f_0}, \beta_{f_0}$$

DataSPM - a table with the following variables (in any order; can have additional variables which will be ignored): *Age* (start of age interval), *AgeNext* (end of age interval), *IndicatorEvent* (a binary variable indicating an event (1) or no event (0) within the age interval (*Age* , *AgeNext*)), *Yt* (longitudinal variable modeled by Y(t,c)), *IsFirstRow* (a binary variable indicating the first record for an individual: 1 - first record; 0 - otherwise), *IsLastRow* (a binary variable indicating the last record for an individual: 1 - last record; 0 - otherwise), and variables to be included as additional covariates (c), see NamesCovar

t_min - minimal age used in formulas, see above

NamesCovar - cell array with names of variables in DataSPM to be used as additional covariates (c): the first cell contains names of variables to be used as covariates in $\mu_0(t,c)$ and the second cell contains names of variables to be used as covariates in other components

Output:

lnLik - minus logarithm of the likelihood function

```
function lnLik = LogLikSPM(Param, DataSPM, t_min, NamesCovar)

NumRows = height(DataSPM);

NamesCovarMu0 = NamesCovar{1};
NamesCovarOther = NamesCovar{2};
NumCovarMu0 = length(NamesCovarMu0);
NumCovarOther = length(NamesCovarOther);

ln_a_mu0 = Param(1);
b_mu0 = Param(2);
b_covar_mu0 = Param(3:(3 + NumCovarMu0 - 1));
a_Q = Param(3 + NumCovarMu0);
b_Q = Param(4 + NumCovarMu0);
b_covar_Q = Param((5 + NumCovarMu0):(5 + NumCovarMu0 + NumCovarOther - 1));
a_Y = Param(5 + NumCovarMu0 + NumCovarOther);
b_Y = Param(6 + NumCovarMu0 + NumCovarOther);
b_covar_Y = Param((7 + NumCovarMu0 + NumCovarOther):(7 + NumCovarMu0 + 2*NumCovarOther - 1));
sigma0 = Param(7 + NumCovarMu0 + 2*NumCovarOther);
sigma1 = Param(8 + NumCovarMu0 + 2*NumCovarOther);
b_covar_W = Param((9 + NumCovarMu0 + 2*NumCovarOther):(9 + NumCovarMu0 + 3*NumCovarOther - 1));
a_f1 = Param(9 + NumCovarMu0 + 3*NumCovarOther);
b_f1 = Param(10 + NumCovarMu0 + 3*NumCovarOther);
b_covar_f1 = Param((11 + NumCovarMu0 + 3*NumCovarOther):(11 + NumCovarMu0 + 4*NumCovarOther - 1));
a_f0 = Param(11 + NumCovarMu0 + 4*NumCovarOther);
b_f0 = Param(12 + NumCovarMu0 + 4*NumCovarOther);
b_covar_f0 = Param((13 + NumCovarMu0 + 4*NumCovarOther):(13 + NumCovarMu0 + 5*NumCovarOther - 1));

delta_i_all = DataSPM.IndicatorDeath;
t = DataSPM.Age;
t_next = DataSPM.AgeNext;
Yt = DataSPM.Yt;
IsFirstRow = DataSPM.IsFirstRow;
IsLastRow = DataSPM.IsLastRow;

if NumCovarOther == 1
    Xt_other = DataSPM.(NamesCovarOther{1});
else
    Xt_other = NaN*ones(NumRows, NumCovarOther);
    for i = 1:NumCovarOther
        Xt_other(:, i) = DataSPM.(NamesCovarOther{i});
    end
end

if NumCovarMu0 == 1
    Xt_mu0 = DataSPM.(NamesCovarMu0{1});
```

```
else
    Xt_mu0 = NaN*ones(NumRows, NumCovarMu0);
    for i = 1:NumCovarMu0
        Xt_mu0(:, i) = DataSPM.(NamesCovarMu0{i});
    end
end

lnLik = 0;
for i = 1:NumRows
    delta_i = delta_i_all(i);
    tk = t(i);
    tk_next = t_next(i);
    Ytk = Yt(i);
    Xtk_other = Xt_other(i, :);
    Xtk_mu0 = Xt_mu0(i, :);

    mu0_tk = exp(ln_a_mu0 + b_mu0*(tk - t_min) + Xtk_mu0*b_covar_mu0);
    f0_tk = a_f0 + b_f0*(tk - t_min) + Xtk_other*b_covar_f0;
    Q_tk = a_Q + b_Q*(tk - t_min) + Xtk_other*b_covar_Q;
    mu_tk = mu0_tk + Q_tk*(Ytk - f0_tk)^2;

    if IsFirstRow(i) == 1
        lnLY = 0;
        lnLQ = 0;

        t0 = t(i);
        Yt0 = Yt(i);

        Ybar_tk_prev = a_f1 + b_f1*(t0 - t_min) + Xtk_other*b_covar_f1;
        if (sigma0 > 0)
            lnLY = lnLY - log(sqrt(2*pi)*sigma0) - ((Yt0 - Ybar_tk_prev)^2)/(2*sigma0^2);
        end
    else
        tk_prev = t(i-1);
        Ytk_prev = Yt(i-1);

        a_tk_prev = a_Y + b_Y*(tk_prev - t_min) + Xtk_other*b_covar_Y;
        f1_tk_prev = a_f1 + b_f1*(tk_prev - t_min) + Xtk_other*b_covar_f1;

        Ybar_tk_prev = Ytk_prev + a_tk_prev*(Ytk_prev - f1_tk_prev)*(tk - tk_prev);
        sigma1_tk = sigma1 + Xtk_other*b_covar_W;
        if (sigma1_tk > 0) && ((tk - tk_prev) > 0)
            lnLY = lnLY - log(sqrt(2*pi*(tk - tk_prev))*sigma1_tk) - ((Ytk - Ybar_tk_prev)^2)/(2*(tk -
tk_prev)*sigma1_tk^2);
        end
    end

    if IsLastRow(i) == 1
        if delta_i == 0
            lnLQ = lnLQ - mu_tk*(tk_next - tk);
        elseif delta_i == 1
            lnLQ = lnLQ + log(1 - exp(-mu_tk*(tk_next - tk)));
        end
    else
        lnLQ = lnLQ - mu_tk*(tk_next - tk);
    end
```

```
    if IsLastRow(i) == 1
        lnLik = lnLik + lnLY + lnLQ;
    end
end
lnLik = -lnLik;
```

Published with MATLAB® R2024b
