# Supplementary material for: Methods for joint modeling of longitudinal omics data and time-to-event outcomes: applications to lysophosphatidylcholines in connection to aging and mortality in the Long Life Family Study
Source: Aging (Albany NY). 2025 May 27;17(5):1221–60. doi: 10.18632/aging.206259 (PMC12151508; doi:10.18632/aging.206259)
Supplement: Supplementary Figures [file aging-17-206259-s002.pdf]

SUPPLEMENTARY FIGURES

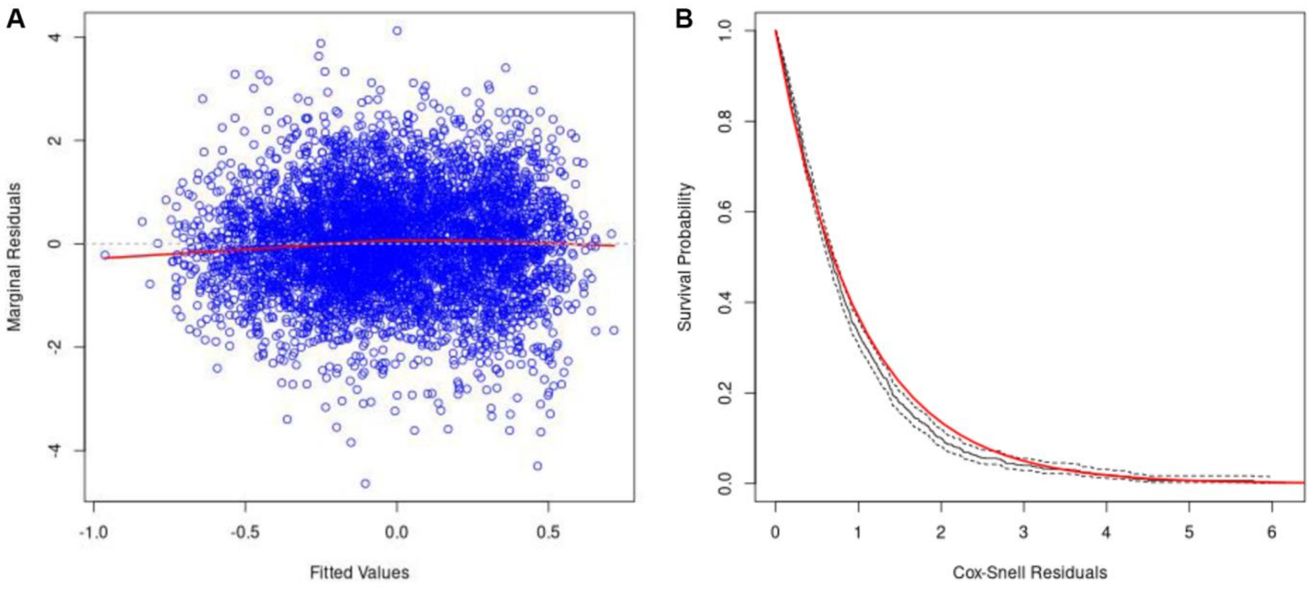

Supplementary Figure 1. Diagnostic plots assessing the goodness-of-fit and assumptions of joint models in applications to LPC 15:0/0:0. (**A**) Plot of the standardized marginal residuals versus the corresponding fitted values for the longitudinal outcome (LPC 15:0/0:0). The red solid line denotes the fit of the loess smoother. (**B**) Residuals analysis for the survival outcome by assessing the overall fit of the survival sub-model using the Cox-Snell residuals. The black solid line denotes the Kaplan-Meier estimate of the survival function of the residuals (with the dashed lines corresponding to the 95% pointwise confidence intervals). The red solid line is the survival function of the unit exponential distribution (this is the distribution if the survival sub-model is correct).

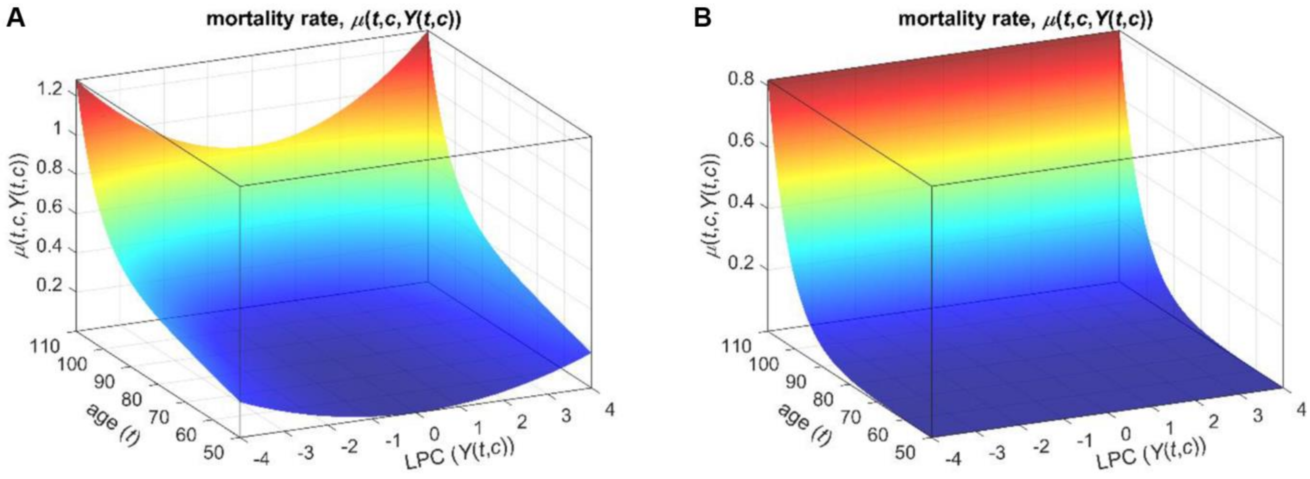

Supplementary Figure 2. Stochastic process models: 3D plots illustrating mortality rates corresponding to different quadratic hazard terms $\boldsymbol{Q}(\boldsymbol{t},\boldsymbol{c})$. (**A**) Mortality rate in the case of non-zero $\boldsymbol{Q}(\boldsymbol{t},\boldsymbol{c})$; (**B**) Mortality rate when $\boldsymbol{Q}(\boldsymbol{t},\boldsymbol{c}) = \boldsymbol{0}$. For this illustration, we modeled Eqs. (6–7) with a single binary covariate c and parameters corresponding to a hypothetical biomarker $\boldsymbol{Y}(\boldsymbol{t},\boldsymbol{c})$ (e.g., a transformed LPC) having the standard normal distribution and hazard rates resembling human mortality rates at old ages. The figures display the mortality rate $\boldsymbol{\mu}(\boldsymbol{t},\boldsymbol{c},\boldsymbol{Y}(\boldsymbol{t},\boldsymbol{c}))$ (Eq. (7)) as a function of age t, covariate c (shown for c = 0), and the hypothetical biomarker $\boldsymbol{Y}(\boldsymbol{t},\boldsymbol{c})$.

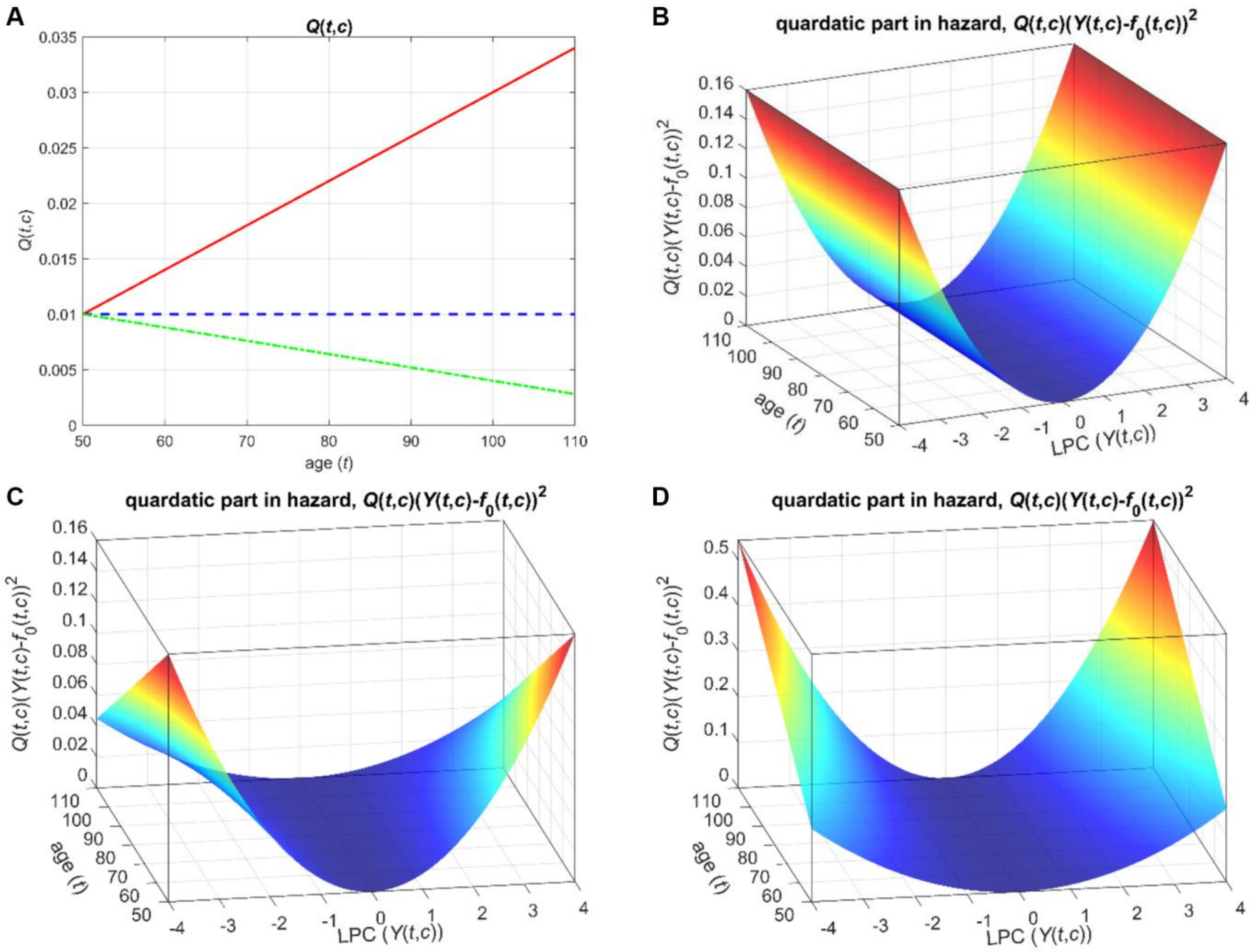

Supplementary Figure 3. Stochastic process models: Illustration of the quadratic part in the hazard corresponding to different age patterns of $\boldsymbol{Q(t,c)}$. (**A**) Age patterns of $\boldsymbol{Q(t,c)}$: age-independent (dashed blue line), declining with age (dash-dotted green line), and increasing with age (solid red line); (**B**) The quadratic part in the hazard in the case of age-independent $\boldsymbol{Q(t,c)}$; (**C**) The quadratic part in the hazard corresponding to $\boldsymbol{Q(t,c)}$ declining with age; (**D**) The quadratic part in the hazard when $\boldsymbol{Q(t,c)}$ is increasing with age. For this illustration, we modeled Eqs. (6–7) with a single binary covariate *c* and parameters corresponding to a hypothetical biomarker $\boldsymbol{Y(t,c)}$ (e.g., a transformed LPC) having the standard normal distribution and hazard rates resembling human mortality rates at old ages. The figures (**B**–**D**) display the quadratic part in the hazard in Eq. (7) (i.e., $\boldsymbol{Q(t,c)\left(Y(t,c)-f_0(t,c)\right)^2}$) as a function of age *t*, covariate *c* (shown for *c* = 0), and the hypothetical biomarker $\boldsymbol{Y(t,c)}$ for respective $\boldsymbol{Q(t,c)}$ modeled as a linear function of age *t* and covariate *c*. We assumed $\boldsymbol{f_0(t,c)=0}$ for simplicity of illustration.

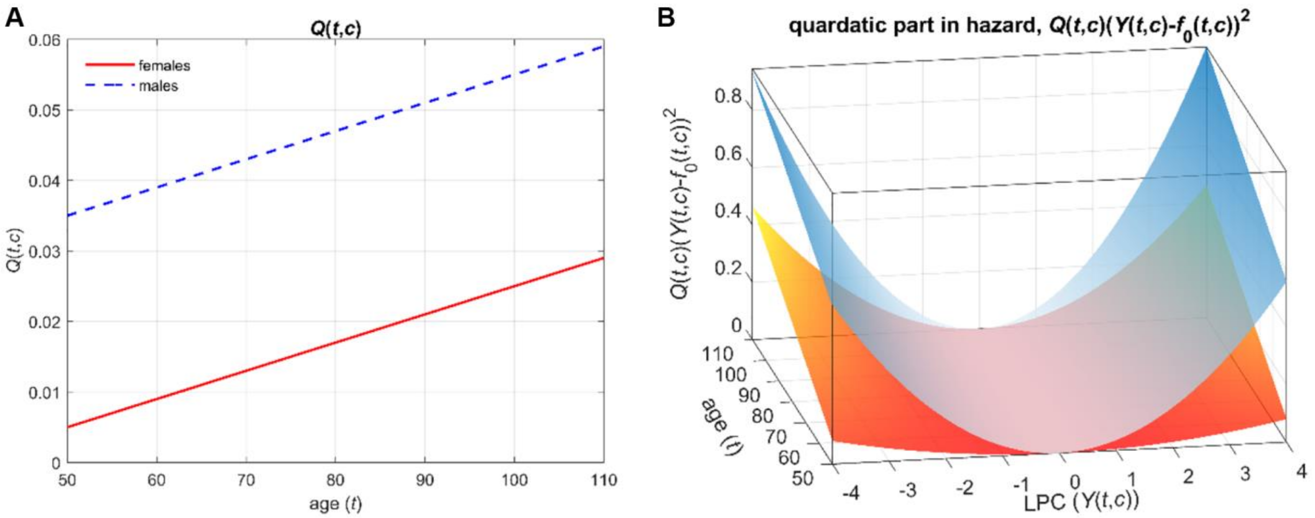

Supplementary Figure 4. Stochastic process models: Illustration of the quadratic part in the hazard corresponding to different patterns of $Q(t,c)$ in females and males. (**A**) Age patterns of $Q(t,c)$ in females (solid red line) and males (dashed blue line); b) The quadratic part in the hazard corresponding to female (surface at the bottom) and male (surface at the top) $Q(t,c)$. For this illustration, we modeled Eqs. (6–7) with a single binary covariate *c* (sex) and parameters corresponding to a hypothetical biomarker $Y(t,c)$ (e.g., a transformed LPC) having the standard normal distribution and hazard rates resembling human mortality rates at old ages. The surfaces in (**B**) show the quadratic part in the hazard in Eq. (7) (i.e., $Q(t,c)\left(Y(t,c)-f_0(t,c)\right)^2$) as a function of age *t*, covariate *c* (*c* = 0 for females, *c* = 1 for males), and the hypothetical biomarker $Y(t,c)$ for respective $Q(t,c)$ modeled as a linear function of age *t* and covariate *c* as shown in Figure A). We assumed $f_0(t,c)=0$ for simplicity of illustration.

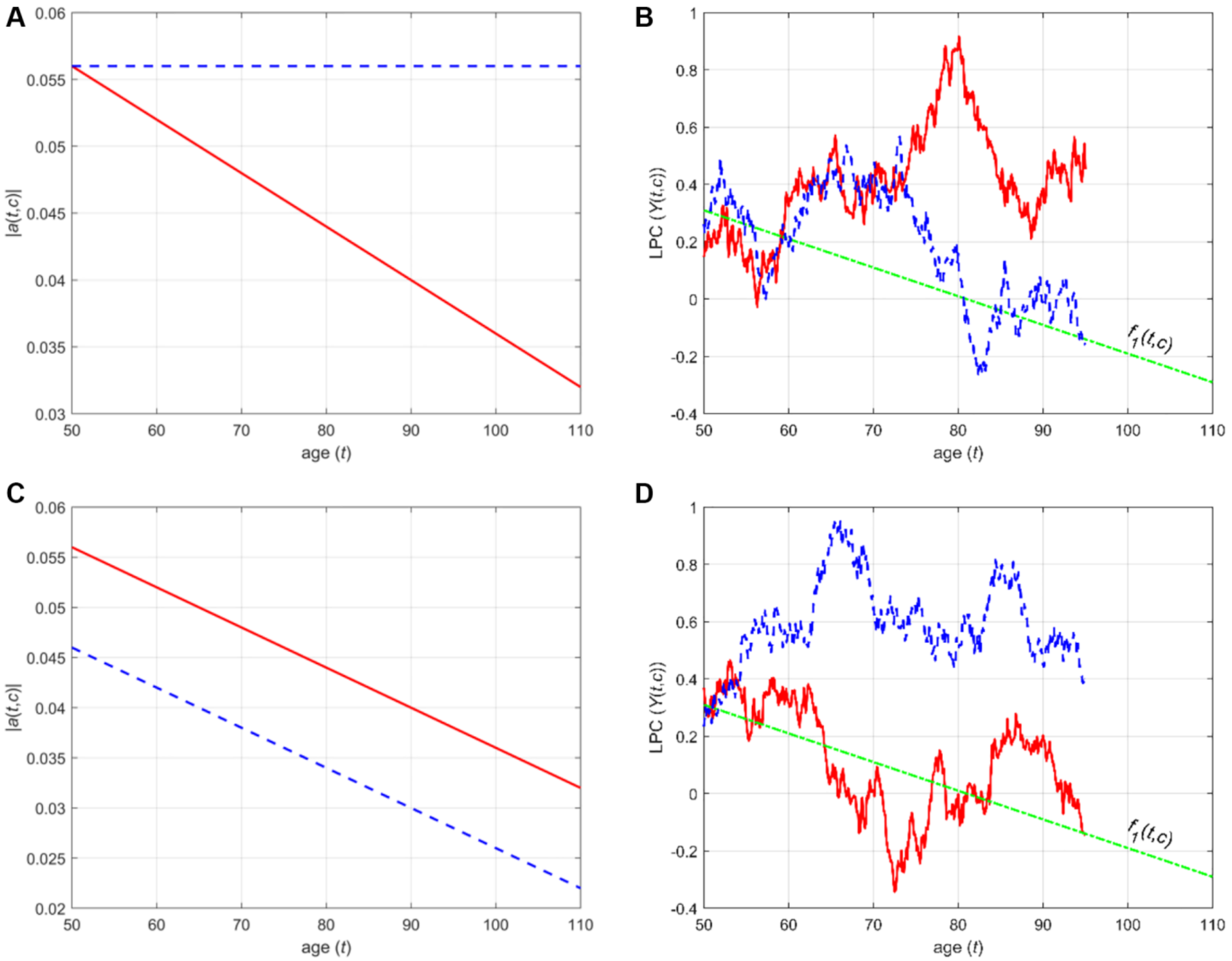

Supplementary Figure 5. Stochastic process models: Illustration of the patterns of the feedback coefficient and sample age trajectories of the process $Y(t,c)$. (**A**) Age-independent (dashed blue line) and declining (solid red line) patterns of the absolute value of the feedback coefficient ($|a(t,c)|$); (**B**) Sample trajectories of $Y(t,c)$ corresponding to respective patterns of $|a(t,c)|$ shown in (A); (**C**) Age patterns of $|a(t,c)|$ for females (solid red line) and males (dashed blue line); (**D**) Sample trajectories of $Y(t,c)$ in females (solid red line) and males (dashed blue line) corresponding to respective patterns of $|a(t,c)|$ shown in (**C**). For this illustration, we modeled Eq. (6) for a hypothetical biomarker $Y(t,c)$ (e.g., a transformed LPC) with respective patterns of the feedback coefficient $a(t,c)$ and a single binary covariate c (sex). The sample trajectories of $Y(t,c)$ shown in (**B**) and (**D**) were generated from respective distributions with feedback coefficients shown in (**A**) and (**C**). We assumed that the equilibrium levels $f_1(t,c)$ (shown as dash-dotted green lines in (**B**) and (**D**)) are similar in both cases, for simplicity of illustration.

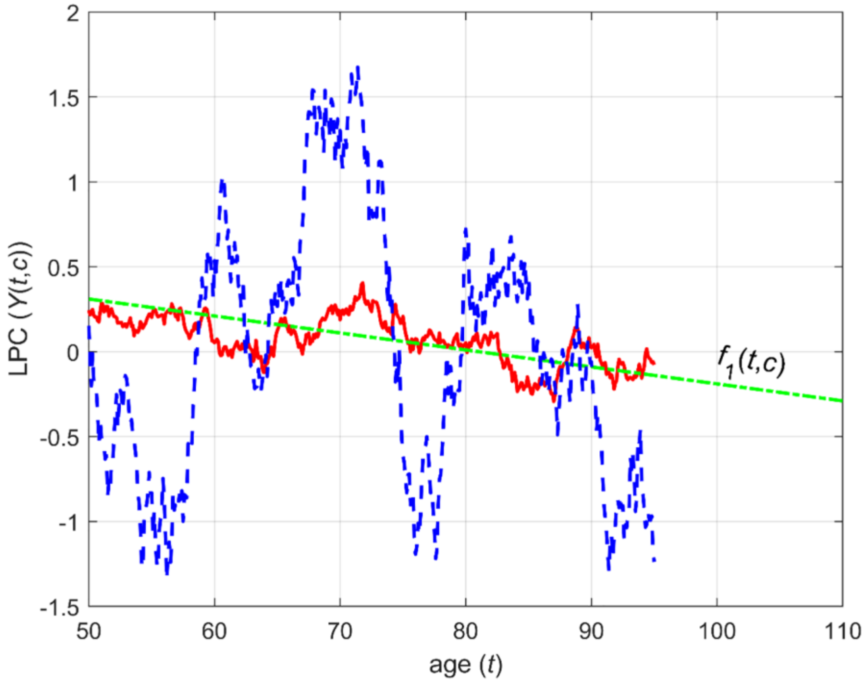

Supplementary Figure 6. Stochastic process models: Examples of trajectories of the stochastic process $Y(t,c)$ with different volatility coefficients. The figure shows sample trajectories of $Y(t,c)$ in the stochastic process models corresponding to higher (dashed blue line) and lower (solid red line) volatility coefficients $b(t,c)$. For this illustration, we modeled Eq. (6) for a hypothetical biomarker $Y(t,c)$ (e.g., a transformed LPC). The sample trajectories of $Y(t,c)$ were generated from respective distributions with different values of the volatility coefficients $b(t,c)$ ($b(t,c) = 0.1$ and $b(t,c) = 0.4$). We assumed that the equilibrium levels $f_1(t,c)$ (shown as the dash-dotted green line) are similar in both cases, for simplicity of illustration.

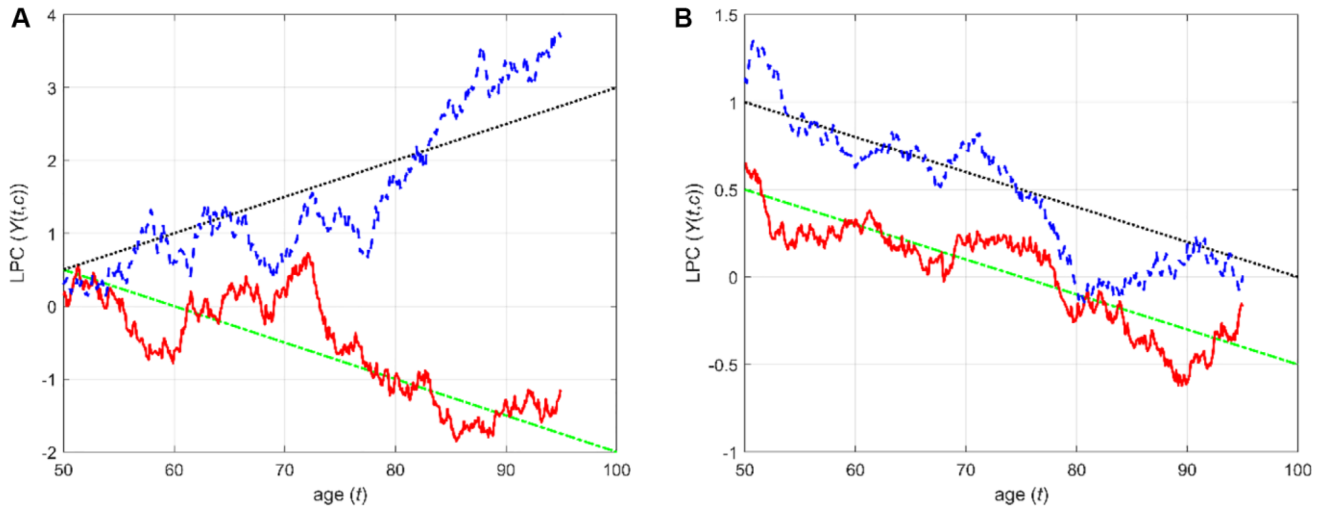

Supplementary Figure 7. Stochastic process models: Examples of trajectories of the stochastic process $Y(t,c)$ with different equilibrium patterns. (**A**) This figure shows two equilibrium trajectories with increasing (black dotted line) and decreasing (green dash-dotted line) age patterns and corresponding sample trajectories of $Y(t,c)$ (blue dashed and red solid lines, respectively); (**B**) This figure shows two equilibrium trajectories shifted by a constant (e.g., representing sex-specific equilibrium levels in the model) (black dotted and green dash-dotted lines) and corresponding sample trajectories of $Y(t,c)$ (blue dashed and red solid lines, respectively). For this illustration, we modeled Eq. (6) for a hypothetical biomarker $Y(t,c)$ (e.g., a transformed LPC) with $f_1(t,c) = a_{f_1} + b_{f_1}(t - t_{min}) + \beta_{f_1}c$ and a single binary covariate c (sex). The sample trajectories of $Y(t,c)$ were generated from respective distributions with different parameters in $f_1(t,c)$ (positive and negative $b_{f_1}$and zero β_{f_1} in (A), negative b_{f_1} and positive β_{f_1} in (B)) so that the black dotted and blue dashed lines correspond to the equilibrium levels and a sample trajectory of $Y(t,c)$ for males).

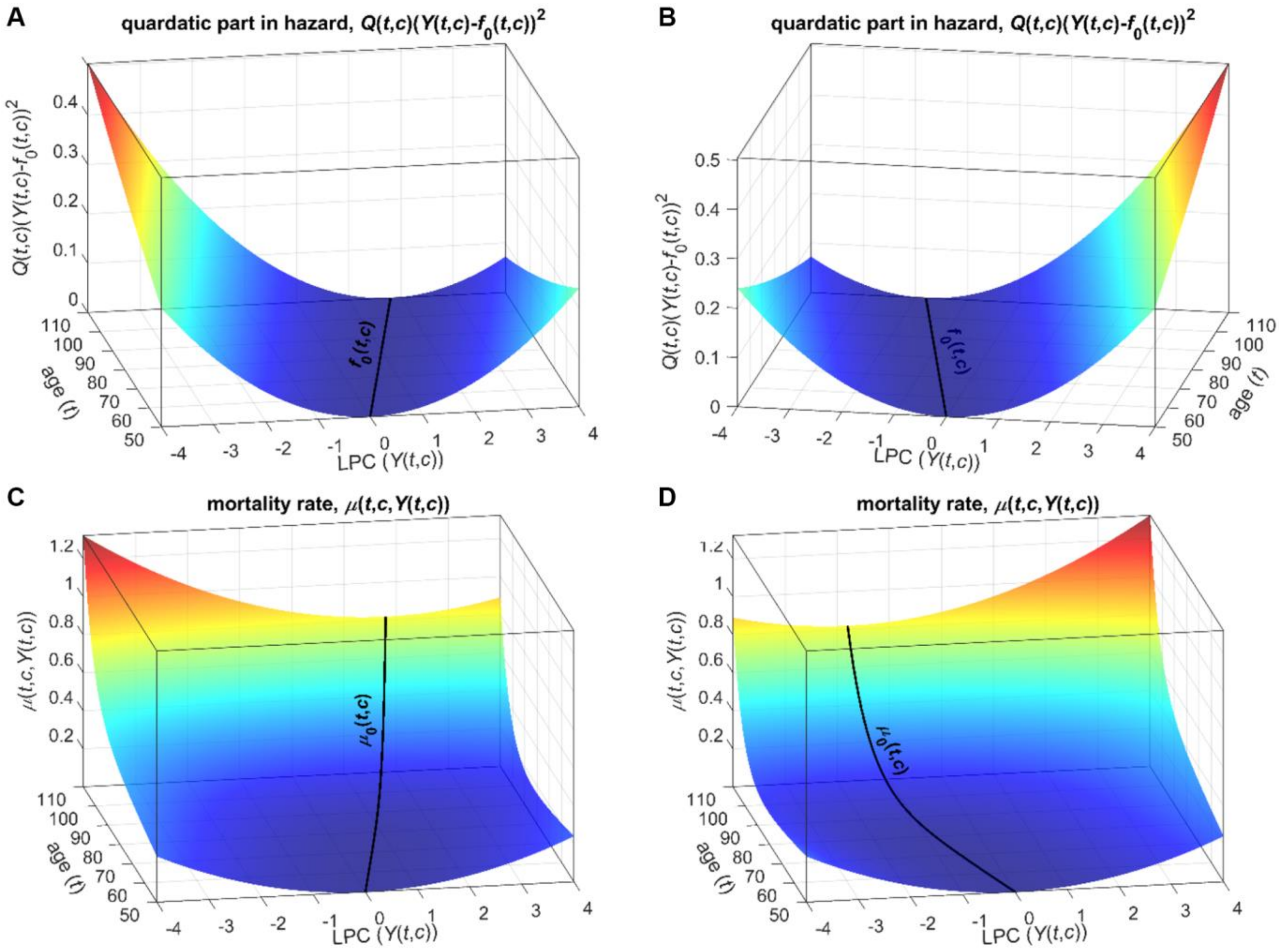

Supplementary Figure 8. Stochastic process models: Illustration of the mortality rate and the quadratic part in the hazard corresponding to different patterns of $\boldsymbol{f_0(t,c)}$. (**A**) The quadratic part in the hazard in the case of the optimal trajectory increasing with age; (**B**) The quadratic part in the hazard corresponding to the optimal trajectory declining with age; (**C**) The mortality rate when the optimal trajectory increases with age; (**D**) The mortality rate when the optimal trajectory declines with age. For this illustration, we modeled Eqs. (6–7) with a single binary covariate *c* and parameters corresponding to a hypothetical biomarker $\boldsymbol{Y(t,c)}$ (e.g., a transformed LPC) having the standard normal distribution and hazard rates resembling human mortality rates at old ages. Figure A, B show the quadratic parts in the hazard from Eq. (7) (i.e., $\boldsymbol{Q(t,c)\left(Y(t,c)-f_0(t,c)\right)^2}$) as a function of age *t*, covariate *c* (shown for *c* = 0), and the hypothetical biomarker $\boldsymbol{Y(t,c)}$ for increasing (**A**) and declining (**B**) $\boldsymbol{f_0(t,c)}$. The figures also display the optimal trajectories $\boldsymbol{f_0(t,c)}$ (black lines) where the quadratic part equals zero so that the mortality rate is $\boldsymbol{\mu_0(t,c)}$ (Eq. (7)). Respective mortality rates $\boldsymbol{\mu\left(t,c,Y(t,c)\right)}$ (Eq. (7)) are presented in (**C**, **D**). These figures also show the baseline mortality $\boldsymbol{\mu_0(t,c)}$ (black lines) observed at the optimal levels $\boldsymbol{f_0(t,c)}$. For simplicity of illustration, we assumed here that $\boldsymbol{Q(t,c)}$ does not depend on age *t* so that the width of the U-shape of mortality as a function of the biomarker $\boldsymbol{Y(t,c)}$ is the same for all ages.

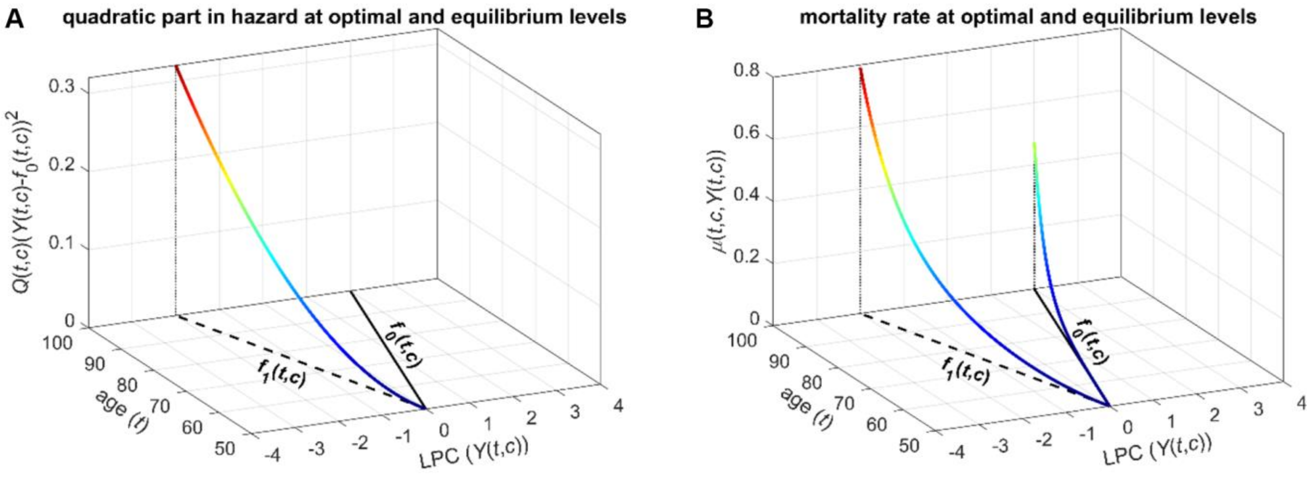

Supplementary Figure 9. Stochastic process models: Illustration of the quadratic part in the hazard and the mortality rate corresponding to equilibrium and optimal trajectories of the stochastic process $Y(t,c)$. The figure shows the quadratic part in the hazard $\boldsymbol{Q(t,c)\big(Y(t,c)-f_0(t,c)\big)^2}$ (**A**) and the mortality rate $\boldsymbol{\mu\big(t,c,Y(t,c)\big)}$ (**B**) from Eq. (7) for different values of age *t*, covariate *c* = 0, and the hypothetical biomarker $\boldsymbol{Y(t,c)}$ evaluated at the optimal ($\boldsymbol{f_0(t,c)}$, solid black lines) and equilibrium ($\boldsymbol{f_1(t,c)}$, dashed black lines) levels for respective ages *t* and *c* = 0. For this illustration, we modeled Eqs. (6)-(7) with a single binary covariate *c* and parameters corresponding to a hypothetical biomarker $\boldsymbol{Y(t,c)}$ (e.g., a transformed LPC) having the standard normal distribution and hazard rates resembling human mortality rates at old ages.

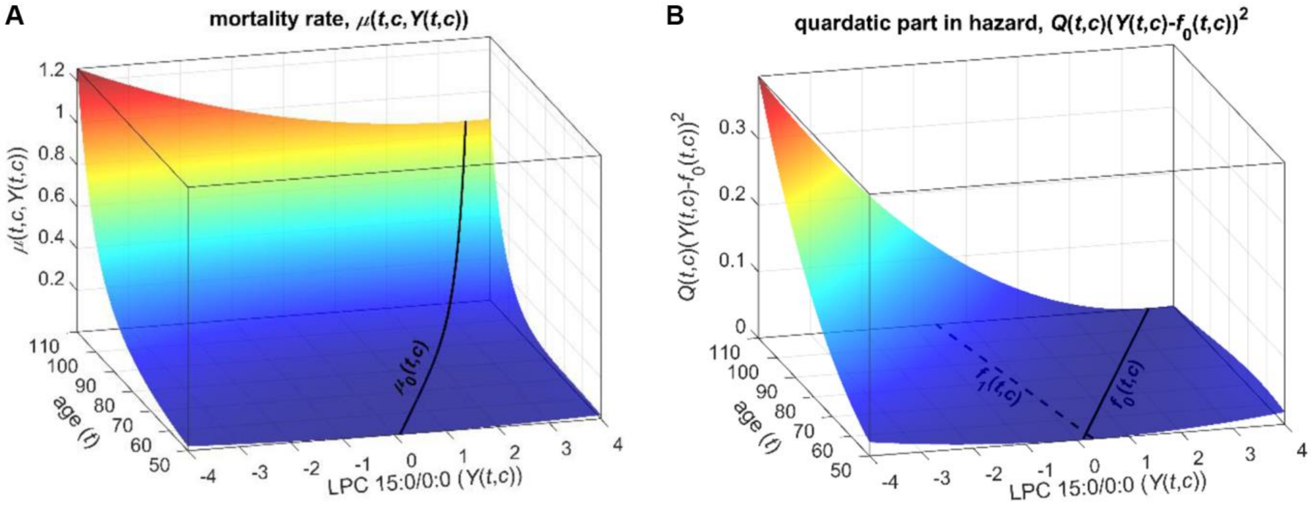

Supplementary Figure 10: Stochastic process models: 3D plots illustrating results of applications to measurements of LPC 15:0/0:0 and mortality data in the LLFS. (**A**) Estimates of the mortality rate ($\boldsymbol{\mu\big(t,c,Y(t,c)\big)}$) as a function of age (*t*), covariates (*c*), and LPC 15:0/0:0 ($\boldsymbol{Y(t,c)}$); (**B**) Estimates of the quadratic part in the equation for the hazard (mortality) rate ($\boldsymbol{\mu\big(t,c,Y(t,c)\big)-\mu_0(t,c)}$) for different ages (*t*) and values of LPC 15:0/0:0 ($\boldsymbol{Y(t,c)}$). The estimates are shown for zero (mean) values of binary (continuous) covariates *c*. The solid black lines denote the baseline mortality rate $\boldsymbol{\mu_0(t,c)}$ (Supplementary Figure 10A) and the optimal trajectory $\boldsymbol{f_0(t,c)}$ (Supplementary Figure 10B). The dashed black line corresponds to the equilibrium trajectory $\boldsymbol{f_1(t,c)}$. LPC values were transformed (see Data).
