# Supplementary material for: Methods for joint modeling of longitudinal omics data and time-to-event outcomes: applications to lysophosphatidylcholines in connection to aging and mortality in the Long Life Family Study
Source: Aging (Albany NY). 2025 May 27;17(5):1221–60. doi: 10.18632/aging.206259 (PMC12151508; doi:10.18632/aging.206259)
Supplement: Supplementary Tables [file aging-17-206259-s003.pdf]

SUPPLEMENTARY TABLES

Supplementary Table 1. Results of applications of joint models with shared random effects to measurements of LPC species and mortality data in the LLFS: Estimates of the association parameters for the random intercepts and random slopes of the metabolite in the survival sub-model.

Model	Metabolite	Variable	α_0 (α_1)	HR	95% CI for HR	SD of Variable
int	LPC 0:0/16:0	b_{0i}	**−0.229**	**0.900**	**(0.845, 0.963)**	**0.461**
intslope	LPC 0:0/16:0	b_{0i}	**−0.403**	**0.828**	**(0.808, 0.948)**	**0.470**
intslope	LPC 0:0/16:0	b_{1i}	−6.115	0.990	(0.969, 1.007)	0.002
int	LPC 0:0/16:1	b_{0i}	−0.010	0.996	(0.931, 1.063)	0.437
intslope	LPC 0:0/16:1	b_{0i}	**−0.080**	**0.965**	**(0.817, 0.951)**	**0.445**
intslope	LPC 0:0/16:1	b_{1i}	−2.120	0.996	(0.967, 1.008)	0.002
int	LPC 0:0/18:0	b_{0i}	−0.025	0.986	(0.920, 1.053)	0.572
intslope	LPC 0:0/18:0	b_{0i}	−0.203	0.888	(0.880, 1.036)	0.585
intslope	LPC 0:0/18:0	b_{1i}	−3.645	0.988	(0.988, 1.010)	0.003
int	LPC 0:0/18:1	b_{0i}	**−0.148**	**0.931**	**(0.875, 0.996)**	**0.486**
intslope	LPC 0:0/18:1	b_{0i}	−0.333	0.849		0.490
intslope	LPC 0:0/18:1	b_{1i}	−16.596	0.989		0.001
int	LPC 0:0/18:2	b_{0i}	**−0.213**	**0.909**	**(0.849, 0.976)**	**0.447**
intslope	LPC 0:0/18:2	b_{0i}	−0.207	0.911		0.450
intslope	LPC 0:0/18:2	b_{1i}	−1.224	0.999		0.001
int	LPC 0:0/20:3	b_{0i}	−0.114	0.950	(0.894, 1.010)	0.455
intslope	LPC 0:0/20:3	b_{0i}	−0.16	0.916		0.547
intslope	LPC 0:0/20:3	b_{1i}	−0.55	0.989		0.020
int	LPC 0:0/20:4	b_{0i}	**−0.158**	**0.922**	**(0.865, 0.984)**	**0.515**
intslope	LPC 0:0/20:4	b_{0i}	**−0.236**	**0.885**	**(0.847, 0.980)**	**0.519**
intslope	LPC 0:0/20:4	b_{1i}	−5.689	0.995	(0.989, 1.001)	0.001
int	LPC 0:0/22:6	b_{0i}	**−0.179**	**0.91**	**(0.847, 0.969)**	**0.529**
intslope	LPC 0:0/22:6	b_{0i}	**−0.17**	**0.901**	**(0.822, 0.965)**	**0.610**
intslope	LPC 0:0/22:6	b_{1i}	−0.155	0.997	(0.893, 1.076)	0.022
int	LPC 14:0/0:0	b_{0i}	**−0.225**	**0.922**	**(0.864, 0.979)**	**0.360**
intslope	LPC 14:0/0:0	b_{0i}	**−0.240**	**0.905**	**(0.846, 0.973)**	**0.416**
intslope	LPC 14:0/0:0	b_{1i}	−0.376	0.996	(0.521, 1.173)	0.012
int	LPC 15:0/0:0	b_{0i}	**−0.436**	**0.786**	**(0.728, 0.840)**	**0.553**
intslope	LPC 15:0/0:0	b_{0i}	**−0.452**	**0.775**	**(0.726, 0.846)**	**0.563**
intslope	LPC 15:0/0:0	b_{1i}	−0.765	0.998	(0.994, 1.004)	0.002
int	LPC 16:0/0:0	b_{0i}	**−0.230**	**0.889**	**(0.835, 0.947)**	**0.511**
intslope	LPC 16:0/0:0	b_{0i}	−0.369	0.825		0.522
intslope	LPC 16:0/0:0	b_{1i}	−3.785	0.990		0.003
int	LPC 16:1/0:0	b_{0i}	−0.034	0.985	(0.920, 1.053)	0.457
intslope	LPC 16:1/0:0	b_{0i}	−0.249	0.890	(0.891, 1.038)	0.468
intslope	LPC 16:1/0:0	b_{1i}	−4.560	0.988	(0.982, 1.030)	0.003
int	LPC 17:0/0:0	b_{0i}	**−0.229**	**0.888**	**(0.832, 0.949)**	**0.518**
intslope	LPC 17:0/0:0	b_{0i}	−0.318	0.846		0.524
intslope	LPC 17:0/0:0	b_{1i}	−4.138	0.994		0.002
int	LPC 18:0/0:0	b_{0i}	0.022	1.014	(0.945, 1.080)	0.620
intslope	LPC 18:0/0:0	b_{0i}	0.014	1.009	(0.927, 1.070)	0.625
intslope	LPC 18:0/0:0	b_{1i}	0.217	1.001	(0.994, 1.013)	0.005

int	LPC 18:1/0:0	b_{0i}	**−0.238**	**0.886**	**(0.831, 0.949)**	**0.506**
intslope	LPC 18:1/0:0	b_{0i}	**−0.430**	**0.801**	**(0.804, 0.944)**	**0.515**
intslope	LPC 18:1/0:0	b_{1i}	−5.637	0.988	(0.985, 1.008)	0.002
int	LPC 18:2/0:0	b_{0i}	**−0.277**	**0.883**	**(0.825, 0.943)**	**0.450**
intslope	LPC 18:2/0:0	b_{0i}	**−0.276**	**0.883**	**(0.817, 0.942)**	**0.452**
intslope	LPC 18:2/0:0	b_{1i}	−1.238	0.999	(0.995, 1.002)	0.001
int	LPC 18:3/0:0	b_{0i}	−0.144	0.953	(0.886, 1.020)	0.336
intslope	LPC 18:3/0:0	b_{0i}	−0.148	0.950		0.346
intslope	LPC 18:3/0:0	b_{1i}	−0.640	0.998		0.003
int	LPC 20:2/0:0	b_{0i}	**−0.221**	**0.904**	**(0.849, 0.969)**	**0.458**
intslope	LPC 20:2/0:0	b_{0i}	**−0.227**	**0.900**	**(0.815, 0.960)**	**0.462**
intslope	LPC 20:2/0:0	b_{1i}	−1.342	0.999	(0.988, 1.002)	0.001
int	LPC 20:3/0:0	b_{0i}	**−0.235**	**0.898**	**(0.842, 0.953)**	**0.458**
intslope	LPC 20:3/0:0	b_{0i}	**−0.248**	**0.891**	**(0.807, 0.947)**	**0.463**
intslope	LPC 20:3/0:0		−1.616	0.998	(0.984, 1.000)	0.001
int	LPC 20:4/0:0	b_{0i}	**−0.190**	**0.912**	**(0.855, 0.974)**	**0.487**
intslope	LPC 20:4/0:0	b_{0i}	**−0.183**	**0.914**	**(0.822, 0.976)**	**0.488**
intslope	LPC 20:4/0:0	b_{1i}	−1.252	0.999	(0.994, 1.005)	0.001
int	LPC 20:5/0:0	b_{0i}	**−0.230**	**0.896**	**(0.841, 0.957)**	**0.478**
intslope	LPC 20:5/0:0	b_{0i}	**−0.254**	**0.885**	**(0.830, 0.959)**	**0.483**
intslope	LPC 20:5/0:0	b_{1i}	−1.573	0.998	(0.994, 1.004)	0.001
int	LPC 22:5/0:0	b_{0i}	**−0.215**	**0.892**	**(0.838, 0.955)**	**0.530**
intslope	LPC 22:5/0:0	b_{0i}	−0.291	0.856		0.536
intslope	LPC 22:5/0:0	b_{1i}	−4.164	0.994		0.001
int	LPC 22:6/0:0	b_{0i}	**−0.210**	**0.892**	**(0.830, 0.951)**	**0.546**
intslope	LPC 22:6/0:0	b_{0i}	**−0.210**	**0.892**	**(0.826, 0.951)**	**0.546**
intslope	LPC 22:6/0:0	b_{1i}	0.369	1.000	(0.999, 1.002)	0.0004

Model – type of joint model (int – random intercept of LPC in survival sub-model, intslope – random intercept and slope of LPC in survival sub-model), see section Joint models: General specifications; Variable – b_{0i}: random intercept of the metabolite, b_{1i}: random slope of the metabolite; α_0 (α_1) – estimates of the regression parameters for b_{0i} (b_{1i}) in respective models; HR – hazard ratios for an increase by a standard deviation of Variable; 95% CI for HR – respective 95% confidence intervals for HRs; SD of Variable – standard deviation of Variable. Highlighted in bold are cases where confidence intervals do not contain one. The JM were estimated using the R-package *joineR*. LPC values were transformed (see section Data). Note that for some LPCs, CIs are not available due to technical issues encountered in applications of the R-package *joineR*.

Supplementary Table 2. Results of applications of the stochastic process model to measurements of LPC species and mortality data in the LLFS: Results of testing different null hypotheses on age patterns of the model's components.

LPC	H0						
	Qzero	QnoT	AnoT	F1noT	F0noT	ALzero	ALnoT
LPC 0:0/16:0	0.001	0.64	0.52	<0.0001	0.87	0.88	<0.0001
LPC 0:0/16:1	0.010	1	0.83	<0.0001	0.79	0.97	<0.0001
LPC 0:0/18:0	0.011	1	0.46	0.0002	0.22	0.59	0.0005
LPC 0:0/18:1	0.049	1	1	<0.0001	0.15	0.54	<0.0001
LPC 0:0/18:2	0.031	1	1	<0.0001	0.13	0.055	<0.0001
LPC 0:0/20:3	0.005	0.48	1	<0.0001	0.015	0.074	<0.0001
LPC 0:0/20:4	0.007	0.20	1	<0.0001	0.013	0.018	<0.0001
LPC 0:0/22:6	0.005	0.34	0.37	<0.0001	0.44	0.009	<0.0001

LPC 14:0/0:0	<0.0001	0.25	0.08	<0.0001	0.31	0.31	<0.0001
LPC 15:0/0:0	<0.0001	0.0002	1	<0.0001	0.0002	<0.0001	<0.0001
LPC 16:0/0:0	<0.0001	0.48	0.78	<0.0001	0.45	0.53	<0.0001
LPC 16:1/0:0	0.003	1	0.48	<0.0001	0.81	0.96	<0.0001
LPC 17:0/0:0	0.005	0.43	1	<0.0001	0.63	0.37	<0.0001
LPC 18:0/0:0	0.033	1	1	0.040	0.14	0.37	0.040
LPC 18:1/0:0	0.007	0.19	1	<0.0001	0.23	0.44	<0.0001
LPC 18:2/0:0	0.008	0.11	1	<0.0001	0.019	0.091	<0.0001
LPC 18:3/0:0	0.044	1	1	<0.0001	0.16	0.082	<0.0001
LPC 20:2/0:0	0.002	1	1	<0.0001	1	0.85	<0.0001
LPC 20:3/0:0	0.002	0.10	0.94	<0.0001	0.09	0.34	<0.0001
LPC 20:4/0:0	0.004	0.20	1	<0.0001	0.011	0.014	<0.0001
LPC 20:5/0:0	0.009	0.27	1	<0.0001	0.39	0.012	<0.0001
LPC 22:5/0:0	0.0008	0.047	1	<0.0001	0.005	0.014	<0.0001
LPC 22:6/0:0	0.007	1	1	<0.0001	0.69	0.017	<0.0001

Notes: The table shows results (p-values) of testing the following null hypotheses (H0) corresponding to one or more restrictions on parameters of SPM (see Stochastic process models: Specific parameterizations used in applications): H0: $Q(t,c) = 0$ (Qzero); H0: $Q(t,c) = Q(c)$ (QnoT); H0: $a(t,c) = a(c)$ (AnoT); H0: $f_1(t,c) = f_1(c)$ (F1noT); H0: $f_0(t,c) = f_0(c)$ (F0noT); H0: $f_1(t,c) = f_0(t,c)$, i.e., $AL(t,c) = 0$ (ALzero); H0: $f_1(t,c) = f_1(c)$ and $f_0(t,c) = f_0(c)$, i.e., $AL(t,c) = AL(c)$ (ALnoT).

Supplementary Table 3. Results of applications of the stochastic process model to measurements of LPC species and mortality data in the LLFS: Results of testing different null hypotheses on sex-dependence of the model's components.

LPC	H0						
	MU0noC	QnoC	AnoC	BnoC	F1noC	F0noC	ALLnoC
LPC 0:0/16:0	<0.0001	0.09	0.57	0.40	0.56	0.57	<0.0001
LPC 0:0/16:1	<0.0001	0.037	0.18	0.75	<0.0001	1	<0.0001
LPC 0:0/18:0	<0.0001	0.10	0.59	0.58	<0.0001	1	<0.0001
LPC 0:0/18:1	<0.0001	0.53	0.61	0.63	0.63	1	<0.0001
LPC 0:0/18:2	<0.0001	0.35	0.40	0.14	<0.0001	1	<0.0001
LPC 0:0/20:3	<0.0001	0.05	0.71	0.83	0.16	1	<0.0001
LPC 0:0/20:4	<0.0001	0.06	0.35	0.87	0.007	1	<0.0001
LPC 0:0/22:6	<0.0001	0.94	0.77	0.51	0.10	0.37	<0.0001
LPC 14:0/0:0	<0.0001	0.008	0.16	0.74	<0.0001	0.29	<0.0001
LPC 15:0/0:0	<0.0001	0.13	0.12	0.40	0.001	0.16	<0.0001
LPC 16:0/0:0	<0.0001	0.07	0.82	0.40	0.07	0.50	<0.0001
LPC 16:1/0:0	<0.0001	0.011	0.18	0.33	<0.0001	1	<0.0001
LPC 17:0/0:0	<0.0001	0.29	0.39	0.66	0.0004	0.54	<0.0001
LPC 18:0/0:0	<0.0001	0.11	0.85	0.32	<0.0001	1	<0.0001
LPC 18:1/0:0	<0.0001	0.08	0.85	0.96	0.83	0.65	<0.0001
LPC 18:2/0:0	<0.0001	0.11	0.36	0.16	<0.0001	0.50	<0.0001
LPC 18:3/0:0	<0.0001	0.38	0.84	0.77	1	1	<0.0001
LPC 20:2/0:0	<0.0001	0.025	0.37	0.66	0.46	1	<0.0001
LPC 20:3/0:0	<0.0001	0.05	0.53	0.14	0.018	0.68	<0.0001
LPC 20:4/0:0	<0.0001	0.041	0.72	0.46	<0.0001	1	<0.0001
LPC 20:5/0:0	<0.0001	0.39	0.31	0.37	0.018	0.36	<0.0001
LPC 22:5/0:0	<0.0001	0.043	0.63	0.26	<0.0001	0.81	<0.0001
LPC 22:6/0:0	<0.0001	0.76	0.42	0.71	0.14	1	<0.0001

The table shows results (p-values) of testing the following null hypotheses (H0) corresponding to one or more restrictions on parameters of SPM (see Stochastic process models: Specific parameterizations used in applications): H0: $\mu_0(t,c)=\mu_0(t)$ (MU0noC); H0: $Q(t,c)=Q(t)$ (QnoC); H0: $a(t,c)=a(t)$ (AnoC); H0: $b(t,c)=b(t)$ (BnoC); H0: $f_1(t,c)=f_1(t)$ (F1noC); H0: $f_0(t,c)=f_0(t)$ (F0noC); H0: $\mu_0(t,c)=\mu_0(t)$ and $Q(t,c)=Q(t)$ and $a(t,c)=a(t)$ and $b(t,c)=b(t)$ and $f_1(t,c)=f_1(c)$ and $f_0(t,c)=f_0(c)$ (ALLnoC). Here, c denotes variable SexM in respective components. Note that there are other covariates in $\mu_0(t,c)$ (see Stochastic process models: Specific parameterizations used in applications), which still remain in the restricted models in MU0noC and ALLnoC; for brevity of notation, we do not show them in the formulae above.

Supplementary Table 4. Results of applications of the stochastic process model to measurements of LPC species and mortality data in the LLFS: Estimates of parameters in different components of the model.

LPC	$\mu_0(t,c)$								
	$\ln a_{\mu_0}$	b_{μ_0}	β_{μ_0} (SexM)	β_{μ_0} (IsDK)	β_{μ_0} (LowEduc)	β_{μ_0} (Smoke100)	β_{μ_0} (MedsLipid)	β_{μ_0} (MedsHtn)	β_{μ_0} (MedsNitro)
LPC 0:0/16:0	−4.681	0.119	0.491	0.077	−0.069	0.204	−0.235	−0.184	0.474
LPC 0:0/16:1	−4.721	0.118	0.499	0.082	−0.063	0.202	−0.225	−0.184	0.468
LPC 0:0/18:0	−4.815	0.117	0.511	0.076	−0.063	0.199	−0.225	−0.181	0.470
LPC 0:0/18:1	−4.928	0.117	0.476	0.070	−0.062	0.205	−0.221	−0.182	0.459
LPC 0:0/18:2	−4.920	0.117	0.481	0.073	−0.063	0.204	−0.221	−0.183	0.461
LPC 0:0/20:3	−4.688	0.117	0.498	0.081	−0.064	0.209	−0.228	−0.187	0.469
LPC 0:0/20:4	−4.631	0.116	0.512	0.074	−0.068	0.209	−0.227	−0.191	0.478
LPC 0:0/22:6	−4.833	0.119	0.470	0.071	−0.062	0.205	−0.217	−0.178	0.462
LPC 14:0/0:0	−4.576	0.120	0.495	0.079	−0.074	0.212	−0.249	−0.191	0.488
LPC 15:0/0:0	−3.580	0.127	0.560	−0.020	−0.117	0.225	−0.384	−0.244	0.629
LPC 16:0/0:0	−4.617	0.120	0.502	0.079	−0.074	0.205	−0.245	−0.184	0.481
LPC 16:1/0:0	−4.707	0.118	0.502	0.082	−0.066	0.201	−0.227	−0.184	0.470
LPC 17:0/0:0	−4.864	0.119	0.477	0.057	−0.064	0.205	−0.232	−0.184	0.469
LPC 18:0/0:0	−4.825	0.117	0.513	0.072	−0.061	0.200	−0.226	−0.182	0.470
LPC 18:1/0:0	−4.314	0.116	0.509	0.098	−0.074	0.219	−0.251	−0.197	0.498
LPC 18:2/0:0	−4.282	0.116	0.509	0.064	−0.071	0.215	−0.272	−0.203	0.501
LPC 18:3/0:0	−4.896	0.117	0.477	0.072	−0.062	0.205	−0.219	−0.182	0.459
LPC 20:2/0:0	−4.758	0.118	0.503	0.071	−0.065	0.203	−0.233	−0.184	0.470
LPC 20:3/0:0	−4.422	0.115	0.503	0.078	−0.073	0.214	−0.252	−0.196	0.490
LPC 20:4/0:0	−4.559	0.117	0.514	0.073	−0.070	0.210	−0.225	−0.188	0.477
LPC 20:5/0:0	−4.396	0.115	0.576	0.133	−0.067	0.219	−0.228	−0.210	0.515
LPC 22:5/0:0	−4.404	0.116	0.542	0.086	−0.070	0.209	−0.242	−0.205	0.503
LPC 22:6/0:0	−4.819	0.118	0.491	0.084	−0.065	0.204	−0.215	−0.182	0.463

Supplementary Table 4. (Continued)

LPC	$\mu_0(t,c)$								$Q(t,c)$		
	β_{μ_0} (MedsDiab)	β_{μ_0} (APOE4)	β_{μ_0} (NoIntPA)	β_{μ_0} (SPPB)	β_{μ_0} (PREV6)	β_{μ_0} (BMI)	β_{μ_0} (PC1)	β_{μ_0} (PC2)	a_Q	b_Q	β_Q
LPC 0:0/16:0	0.353	0.204	0.032	−0.140	0.191	−0.024	−0.304	−0.125	1.1E-03	4.8E-06	−1.1E-03
LPC 0:0/16:1	0.349	0.203	0.034	−0.140	0.183	−0.023	−0.239	−0.093	1.1E-03	3.3E-11	−1.1E-03
LPC 0:0/18:0	0.354	0.206	0.031	−0.141	0.191	−0.024	−0.158	−0.044	7.9E-04	6.2E-12	−7.9E-04
LPC 0:0/18:1	0.340	0.204	0.034	−0.140	0.199	−0.024	−0.077	0.131	8.8E-05	2.4E-12	−8.8E-05
LPC 0:0/18:2	0.340	0.205	0.032	−0.139	0.200	−0.024	−0.096	0.103	1.1E-04	6.2E-15	−1.1E-04
LPC 0:0/20:3	0.353	0.205	0.035	−0.141	0.199	−0.024	−0.228	−0.150	4.2E-04	1.2E-05	−4.2E-04

LPC 0:0/20:4	0.357	0.211	0.040	−0.145	0.215	−0.025	−0.241	−0.123	5.7E-04	2.4E-05	−5.7E-04
LPC 0:0/22:6	0.354	0.214	0.031	−0.139	0.193	−0.024	−0.201	−0.050	2.0E-04	-3.4E-06	2.0E-05
LPC 14:0/0:0	0.364	0.219	0.038	−0.142	0.210	−0.027	−0.340	−0.319	1.3E-03	1.2E-05	−1.3E-03
LPC 15:0/0:0	0.424	0.286	0.013	−0.168	0.181	−0.046	−0.799	−0.869	1.2E-03	9.6E-05	−1.1E-03
LPC 16:0/0:0	0.356	0.205	0.031	−0.141	0.185	−0.025	−0.372	−0.160	1.3E-03	1.7E-05	−1.3E-03
LPC 16:1/0:0	0.348	0.200	0.033	−0.140	0.185	−0.023	−0.245	−0.131	1.2E-03	1.1E-11	−1.2E-03
LPC 17:0/0:0	0.347	0.207	0.036	−0.140	0.190	−0.024	−0.178	0.058	4.0E-04	6.0E-06	−4.0E-04
LPC 18:0/0:0	0.353	0.206	0.031	−0.142	0.195	−0.024	−0.124	−0.007	5.8E-04	4.7E-12	−5.8E-04
LPC 18:1/0:0	0.349	0.205	0.039	−0.148	0.201	−0.030	−0.389	−0.237	6.9E-04	2.7E-05	−6.9E-04
LPC 18:2/0:0	0.348	0.210	0.044	−0.149	0.219	−0.030	−0.377	−0.311	6.6E-04	3.6E-05	−6.6E-04
LPC 18:3/0:0	0.343	0.204	0.031	−0.139	0.198	−0.024	−0.102	0.087	9.9E-05	1.0E-12	−9.9E-05
LPC 20:2/0:0	0.342	0.201	0.030	−0.140	0.193	−0.024	−0.219	−0.057	1.0E-03	5.0E-12	−1.0E-03
LPC 20:3/0:0	0.362	0.202	0.047	−0.149	0.215	−0.028	−0.313	−0.216	5.6E-04	3.8E-05	−5.6E-04
LPC 20:4/0:0	0.363	0.208	0.035	−0.145	0.210	−0.025	−0.304	−0.198	5.4E-04	2.4E-05	−5.4E-04
LPC 20:5/0:0	0.348	0.207	0.041	−0.153	0.203	−0.032	−0.318	−0.105	3.6E-04	3.9E-05	−3.6E-04
LPC 22:5/0:0	0.351	0.209	0.043	−0.150	0.219	−0.029	−0.286	−0.293	7.0E-04	4.0E-05	−7.0E-04
LPC 22:6/0:0	0.347	0.205	0.030	−0.139	0.195	−0.024	−0.185	−0.016	2.2E-04	1.5E-11	−2.2E-04

Supplementary Table 4. (Continued)

LPC	$a(t,c)$			$Y(t_0,c)$	$b(t,c)$		$f_1(t,c)$			$f_0(t,c)$		
	a_Y	b_Y	β_Y	σ_0	σ_1	β_W	a_{f_1}	b_{f_1}	β_{f_1}	a_{f_0}	b_{f_0}	β_{f_0}
LPC 0:0/16:0	−0.075	1.6E-04	2.0E-04	1.002	0.305	9.3E-03	0.237	−0.012	−0.018	0.200	0.005	2.4E+00
LPC 0:0/16:1	−0.077	5.4E-05	7.9E-03	0.982	0.326	3.7E-03	0.252	−0.007	−0.256	0.154	−0.011	−2.1E-01
LPC 0:0/18:0	−0.063	1.7E-05	2.9E-03	1.006	0.302	−5.9E-03	0.170	−0.004	−0.146	0.298	−0.050	4.9E-01
LPC 0:0/18:1	−0.063	4.9E-10	−3.0E-03	0.987	0.314	5.5E-03	0.198	−0.010	−0.015	4.000	−0.133	−2.6E+00
LPC 0:0/18:2	−0.064	5.9E-13	−5.1E-03	0.952	0.304	1.6E-02	0.233	−0.017	0.188	4.000	−0.133	−2.4E+00
LPC 0:0/20:3	−0.075	7.6E-09	2.1E-03	0.991	0.317	−2.4E-03	0.223	−0.011	−0.044	−1.764	0.096	−7.1E-06
LPC 0:0/20:4	−0.064	2.7E-10	5.2E-03	0.992	0.297	−1.8E-03	0.181	−0.012	0.086	−1.240	0.087	4.5E-06
LPC 0:0/22:6	−0.052	7.5E-10	−1.5E-03	0.989	0.284	6.8E-03	0.139	−0.005	−0.054	2.912	−0.068	−3.4E+00
LPC 14:0/0:0	−0.089	4.7E-04	−4.9E-03	0.980	0.326	−3.8E-03	0.343	−0.013	−0.184	0.242	0.026	1.8E+00
LPC 15:0/0:0	−0.057	4.8E-10	−8.1E-03	0.986	0.276	8.3E-03	0.314	−0.015	−0.104	0.111	0.057	4.8E-01
LPC 16:0/0:0	−0.069	6.7E-05	1.3E-03	1.005	0.298	9.2E-03	0.246	−0.011	−0.057	0.088	0.020	1.1E+00
LPC 16:1/0:0	−0.077	1.8E-04	7.9E-03	0.984	0.316	1.3E-02	0.240	−0.006	−0.278	−0.005	0.009	−3.0E-01
LPC 17:0/0:0	−0.066	1.1E-10	1.8E-03	1.016	0.298	−4.7E-03	0.163	−0.005	−0.115	1.579	−0.033	1.5E+00
LPC 18:0/0:0	−0.049	2.9E-09	−1.0E-03	0.996	0.299	−1.1E-02	0.141	−0.002	−0.145	0.441	−0.074	5.5E-01
LPC 18:1/0:0	−0.064	1.4E-10	1.1E-03	0.987	0.308	5.6E-04	0.232	−0.012	−0.007	−0.532	0.068	4.6E-01
LPC 18:2/0:0	−0.065	1.4E-10	−5.4E-03	0.953	0.301	1.5E-02	0.222	−0.017	0.222	−0.848	0.070	6.2E-01
LPC 18:3/0:0	−0.082	3.4E-10	1.2E-03	0.967	0.331	−3.4E-03	0.250	−0.013	0.000	4.000	−0.133	−2.2E+00
LPC 20:2/0:0	−0.071	1.8E-10	5.2E-03	1.011	0.314	5.0E-03	0.160	−0.008	−0.024	0.323	0.001	−7.0E-02
LPC 20:3/0:0	−0.074	2.0E-05	3.7E-03	0.980	0.307	1.7E-02	0.242	−0.015	0.074	−0.970	0.076	3.8E-01
LPC 20:4/0:0	−0.060	3.7E-11	1.9E-03	0.994	0.288	7.6E-03	0.121	−0.011	0.175	−1.516	0.092	2.4E-06
LPC 20:5/0:0	−0.048	4.0E-11	−5.0E-03	0.975	0.271	8.7E-03	0.241	−0.013	0.075	0.420	0.060	−8.0E-01
LPC 22:5/0:0	−0.065	5.4E-12	2.8E-03	0.983	0.301	1.2E-02	0.108	−0.008	0.142	−0.913	0.082	−1.7E-01
LPC 22:6/0:0	−0.055	3.5E-11	4.3E-03	0.985	0.286	3.9E-03	0.120	−0.006	0.049	2.632	−0.058	−1.8E+00

Notes: Columns $\beta_{\mu_0}(.)$ show coefficients for the variables in the baseline hazard rate ($\mu_0(t,c)$): SexM: sex (1 – male, 0 – female); IsDK: country (1 – Denmark, 0 – USA); LowEduc: low education (1 – below high school, 0 – otherwise); Smoke100: smoking (1 – smoked 100 cigarettes in lifetime, 0 - otherwise); MedsLipid: lipid-lowering medications (1 – taking, 0 – not taking); MedsHtn: hypertension medications (1 – taking, 0 – not taking); MedsNitro: angina medications (1 – taking, 0 – not taking); MedsDiab: diabetes mellitus medications (1 – taking,

0 – not taking); APOE4: *APOE* ε4 carrier status (1 – carrier, 0 – non-carrier); NoIntPA: no intense physical activity (PA) at baseline (1 – no intense PA, 0 – intense PA); SPPB: Short Physical Performance Battery (SPPB) total score at baseline; PREV6: prevalence of major diseases (heart disease, stroke, lung disease, cancer, hypertension, diabetes) at baseline (1: any of the diseases, 0 – none of the diseases); BMI: body mass index (BMI) at baseline; PC1, PC2: first two principal components computed from LLFS whole-genome sequencing data. In other components, respective β's show coefficients for variable SexM. LPC values were transformed (see Data).

Supplementary Table 5. Applications of joint models to measurements of LPC variants and mortality data in the LLFS: Estimates from familial bootstrap.

Metabolite	Median HR (Range)		
	Total	Females	Males
LPC 0:0/16:0	**0.830 (0.743, 0.934)**	**0.843 (0.723, 0.971)**	**0.813 (0.694, 0.968)**
LPC 0:0/16:1	0.961 (0.842, 1.079)	0.989 (0.844, 1.134)	0.935 (0.775, 1.074)
LPC 0:0/18:0	0.977 (0.896, 1.075)	1.003 (0.868, 1.142)	0.942 (0.817, 1.070)
LPC 0:0/18:1	**0.884 (0.796, 0.994)**	0.923 (0.790, 1.083)	**0.831 (0.685, 0.995)**
LPC 0:0/18:2	**0.824 (0.718, 0.942)**	0.910 (0.749, 1.090)	**0.735 (0.611, 0.897)**
LPC 0:0/20:3	**0.852 (0.764, 0.973)**	**0.858 (0.735, 0.976)**	**0.836 (0.691, 0.993)**
LPC 0:0/20:4	**0.860 (0.778, 0.962)**	**0.852 (0.747, 0.986)**	**0.867 (0.736, 0.981)**
LPC 0:0/22:6	**0.842 (0.757, 0.921)**	**0.799 (0.677, 0.900)**	**0.869 (0.735, 0.986)**
LPC 14:0/0:0	**0.828 (0.738, 0.947)**	**0.858 (0.723, 0.995)**	**0.801 (0.670, 0.982)**
LPC 15:0/0:0	**0.713 (0.643, 0.778)**	**0.732 (0.638, 0.850)**	**0.690 (0.591, 0.772)**
LPC 16:0/0:0	**0.831 (0.768, 0.915)**	**0.848 (0.732, 0.998)**	**0.815 (0.739, 0.934)**
LPC 16:1/0:0	0.951 (0.836, 1.056)	0.981 (0.828, 1.135)	0.924 (0.794, 1.044)
LPC 17:0/0:0	**0.821 (0.737, 0.913]**	**0.828 (0.712, 0.955)**	**0.817 (0.705, 0.973)**
LPC 18:0/0:0	1.014 (0.913, 1.115)	1.054 (0.931, 1.214)	0.976 (0.831, 1.142)
LPC 18:1/0:0	**0.820 (0.742, 0.924)**	0.863 (0.731, 1.043)	**0.794 (0.666, 0.932)**
LPC 18:2/0:0	**0.785 (0.678, 0.871)**	0.870 (0.711, 1.049)	**0.707 (0.563, 0.838)**
LPC 18:3/0:0	0.888 (0.756, 1.025)	0.987 (0.855, 1.240)	**0.780 (0.625, 0.985)**
LPC 20:2/0:0	**0.820 (0.715, 0.910)**	0.856 (0.765, 1.048)	**0.774 (0.659, 0.869)**
LPC 20:3/0:0	**0.767 (0.680, 0.873)**	**0.798 (0.657, 0.950)**	**0.746 (0.603, 0.882)**
LPC 20:4/0:0	**0.861 (0.764, 0.949)**	0.897 (0.785, 1.049)	0.808 (0.684, 1.004)
LPC 20:5/0:0	**0.776 (0.696, 0.870)**	**0.742 (0.619, 0.864)**	**0.810 (0.698, 0.989)**
LPC 22:5/0:0	**0.793 (0.714, 0.857)**	**0.781 (0.670, 0.883)**	**0.796 (0.680, 0.898)**
LPC 22:6/0:0	**0.805 (0.733, 0.892)**	**0.759 (0.674, 0.912)**	**0.829 (0.731, 0.936)**

Notes: The table reports medians of hazard ratios (HR) for a unit increase in the transformed metabolite values (ranges in parentheses) for the association parameters for the metabolites in the survival sub-model computed using the familial bootstrap method [18] in 100 bootstrap samples generated from the original sample. See main text (section Materials and Methods: Sensitivity analyses) for details. The joint models were estimated using the R-package *JM*. The cases where the range of HR does not contain one are highlighted in bold. The case where 95% confidence intervals (CI) for HR in the main calculations (Table 1) did not contain one but the HR range in the familial bootstrap included one is highlighted with a yellow background. The cases where 95% CI for HR in the main calculations (Table 1) contained one but the HR range in the familial bootstrap did not include one are highlighted with a grey background.

Supplementary Table 6. Characteristics of the Long Life Family Study metabolomics sample (batch 6, released on October 25, 2023)

Characteristics	Field Center				Total Sample
	BU	NY	PT	DK	
Number of families	244	263	222	77	582
Number of participants at any visit	1,282	875	1,185	1,239	4,581
Number of participants at visit 1	1,176	718	1,131	1,196	4,221

Number of participants at visit 2	682	490	585	798	2,555
Number of participants with genetic PCs	1,270	847	1,174	1,229	4,520
Number (%) of deceased participants	435 (33.9%)	358 (40.9%)	477 (40.3%)	388 (31.3%)	1,658 (36.2%)
Follow-up period (years) (mean ± SD (range))	10.5 ± 4.8 (0.00, 18.22)	10.1 ± 4.5 (0.00, 18.41)	10.8 ± 4.8 (0.29, 18.50)	11.7 ± 5.5 (0.00, 17.93)	10.8 ± 5.0 (0.00, 18.50)
Age at baseline (mean ± SD (range))	69.5 ± 16.0 (32, 110)	73.5 ± 16.1 (24, 108)	71.2 ± 15.9 (36, 104)	67.3 ± 14.3 (36, 104)	70.0 ± 15.7 (24, 110)
Whites (%)	99.2%	98.3%	99.6%	99.0%	99.1%
Females (%)	55.5%	54.6%	55.9%	54.3%	55.1%
Low educated participants (below high school) (%)	5.9%	7.4%	7.2%	27.7%	12.4%
Smokers (smoked >100 cigarettes in lifetime) (%)	41.5%	45.5%	35.6%	49.2%	42.8%
APOE ε4 allele carriers (%)	13.7%	17.3%	17.2%	25.3%	18.4%
Medication use: angina (%)	32.1%	30.4%	32.8%	23.5%	29.6%
Medication use: anti-diabetic (%)	6.8%	7.5%	9.1%	5.5%	7.2%
Medication use: anti-hypertensive (%)	50.4%	52.5%	55.1%	42.0%	49.7%
Medication use: lipid-lowering (%)	34.9%	43.3%	39.2%	21.1%	33.9%
No intense physical activity at baseline (%)	64.2%	56.9%	62.3%	74.0%	65.0%
SPPB total score at baseline (mean ± SD (range))	10.0 ± 3.0 (0, 12)	9.6 ± 3.0 (0, 12)	9.8 ± 3.0 (1, 12)	10.4 ± 2.8 (1, 12)	10.0 ± 3.0 (0, 12)
Prevalence of major diseases at baseline (%)	68.9%	70.4%	69.8%	66.5%	68.8%
BMI at baseline (mean ± SD (range))	27.5 ± 5.1 (16, 57)	26.6 ± 4.2 (17, 41)	27.7 ± 5.2 (17, 52)	26.4 ± 4.2 (13, 54)	27.1 ± 4.8 (13, 57)

Notes: (a) Genetic PCs were computed from LLFS whole-genome sequencing data; (b) Number of missing data: race – 20, education – 10, smoking – 18, *APOE* – 257, angina medications – 273, anti-diabetic drugs – 273, anti-hypertensive drugs – 273, lipid-lowering drugs – 273, no intense physical activity at baseline – 449, SPPB total score at baseline – 179, prevalence of major diseases at baseline – 2, other variables listed in the table have no missing values; (c) The numbers shown in "Number of deceased participants" and "Follow-up period" correspond to the LLFS data release used in this paper (see Data). Abbreviations: BMI: body mass index; BU: Boston; DK: Denmark; NY: New York; PT: Pittsburgh; SD: standard deviation; SPPB: Short Physical Performance Battery.
